# A design–build–test cycle using modeling and experiments reveals interdependencies between upper glycolysis and xylose uptake in recombinant *S. cerevisiae* and improves predictive capabilities of large-scale kinetic models

**DOI:** 10.1186/s13068-017-0838-5

**Published:** 2017-06-26

**Authors:** Ljubisa Miskovic, Susanne Alff-Tuomala, Keng Cher Soh, Dorothee Barth, Laura Salusjärvi, Juha-Pekka Pitkänen, Laura Ruohonen, Merja Penttilä, Vassily Hatzimanikatis

**Affiliations:** 1 Ecole Polytechnique Federale de Lausanne (EPFL), 1015 Lausanne, Switzerland; 2 VTT Technical Research Centre of Finland Ltd, Espoo, Finland

**Keywords:** Bioethanol, Metabolic control analysis, Large-scale kinetic models, Hexokinase, *HXK2* deletion, Xylose utilization, *S. cerevisiae*

## Abstract

**Background:**

Recent advancements in omics measurement technologies have led to an ever-increasing amount of available experimental data that necessitate systems-oriented methodologies for efficient and systematic integration of data into consistent large-scale kinetic models. These models can help us to uncover new insights into cellular physiology and also to assist in the rational design of bioreactor or fermentation processes. Optimization and Risk Analysis of Complex Living Entities (ORACLE) framework for the construction of large-scale kinetic models can be used as guidance for formulating alternative metabolic engineering strategies.

**Results:**

We used ORACLE in a metabolic engineering problem: improvement of the xylose uptake rate during mixed glucose–xylose consumption in a recombinant *Saccharomyces cerevisiae* strain. Using the data from bioreactor fermentations, we characterized network flux and concentration profiles representing possible physiological states of the analyzed strain. We then identified enzymes that could lead to improved flux through xylose transporters (XTR). For some of the identified enzymes, including hexokinase (HXK), we could not deduce if their control over XTR was positive or negative. We thus performed a follow-up experiment, and we found out that *HXK2* deletion improves xylose uptake rate. The data from the performed experiments were then used to prune the kinetic models, and the predictions of the pruned population of kinetic models were in agreement with the experimental data collected on the *HXK2*-deficient *S. cerevisiae* strain.

**Conclusions:**

We present a design–build–test cycle composed of modeling efforts and experiments with a glucose–xylose co-utilizing recombinant *S. cerevisiae* and its *HXK2*-deficient mutant that allowed us to uncover interdependencies between upper glycolysis and xylose uptake pathway. Through this cycle, we also obtained kinetic models with improved prediction capabilities. The present study demonstrates the potential of integrated “modeling and experiments” systems biology approaches that can be applied for diverse applications ranging from biotechnology to drug discovery.

**Electronic supplementary material:**

The online version of this article (doi:10.1186/s13068-017-0838-5) contains supplementary material, which is available to authorized users.

## Background

Nature provides abundant plant biomass that can be potentially converted into biofuels as a promising sustainable source of energy [1, 2]. The main sugar constituents of biomass are stored in the form of hexoses, primarily glucose, and pentoses, primarily xylose. *Saccharomyces cerevisiae,* which is widely used in industry for production of a variety of chemicals, including bioethanol from glucose, is a promising candidate for bioethanol production from sugars. However, the native strain of this organism is unable to utilize xylose because of the lack of a mechanism converting xylose into metabolic intermediates [3, 4].

In efforts to enable xylose utilization in *S. cerevisiae*, the wild-type strains have been modified in two ways: (1) by introducing heterologous *XYL1* and *XYL2* genes from *Scheffersomyces stipitis* (previously *Pichia stipitis*) [5] or *XYL1* from *Candida tenuis* and XYL2 from *Galactocandida mastotermitis* [6, 7] that encode for xylose reductase (XR) and xylitol dehydrogenase (XDH), respectively, which enable the two-step transformation from xylose to xylulose; (2) by expressing a heterologous xylose isomerase (XI) from fungi such as *Piromyces* [8] and *Orpinomyces* [9], or from *Clostridium phytofermentans* [10] which converts xylose to xylulose in one step. Comparison of the two engineered pathways in the same background strain revealed that strains with XR/XDH pathway show higher xylose uptake rates under anaerobic conditions and better aerobic growth [11]. In contrast, it appears that strains with XI have higher ethanol yields due to better cofactor balancing as compared to XR/XDH strains.

While the genetically engineered XR/XDH yeast strains successfully acquired the capability of utilizing xylose as a carbon source, a number of factors cause the suboptimal performance of bioconversion from xylose to bioethanol: low transport efficiency of xylose across the cellular membrane through hexose transporters; different cofactor specificities of XR and XDH reactions; high accumulation of xylitol; and excessive activity of the oxidative pentose phosphate pathway [12].

In an attempt to improve xylose uptake rate and obtain a high ethanol yield from xylose fermentation using recombinant *S. cerevisiae* strains, a number of metabolic engineering efforts have been carried out targeting different enzymes of the metabolic network [13]. Some of the enzymes that have been investigated include hexose transporters (GTR and XTR) [14–17], xylose reductase (XR) [18, 19], xylulose kinase (XK) [20], xylitol dehydrogenase (XDH) [21], and glucose-6-phosphate-1-dehydrogenase (ZWF) [22]. More recently, alteration of the cofactor specificity of XR and XDH has become an important part of efforts to alleviate the cofactor imbalance and to reduce production of xylitol [7, 22, 23]. Despite their sophisticated genetic design, these approaches have not achieved yet commercially viable improvements in the utilization of xylose sugar and its subsequent conversion to ethanol. Therefore, it appears that metabolic engineering strategies stemming from a systems-level analysis of cellular pathways can play an important role in creating potential breakthroughs and advancing the research in this area.

Mathematical methods are well suited to address complex metabolic engineering problems that lack extensive experimental data. These methods allow quantification of the impact of individual enzymes on the overall performance of the metabolic network through response analysis of metabolic fluxes with respect to various enzymatic modifications [24]. A mathematical framework named metabolic control analysis (MCA) has been introduced for this purpose [25]. As an outcome of this analysis, the enzymes whose activity has greater impact on desired metabolic flux rates (e.g., xylose uptake) could be identified and they can be considered as high-priority targets for metabolic engineering.

Kinetic models of xylose metabolism have been proposed by different groups previously [26–28]. Eliasson et al. built a four-reaction model (XRI, XRII, XDH, and XK) to find a XR/XDH/XK ratio that minimizes xylitol formation during xylose utilization [26]. Parachin et al. constructed models for XR/XDH and XI xylose uptake pathways starting with xylose transporters and ending with XK, and found that in both cases increased XK activity led to improved xylose utilization [27]. Recently, Trausinger et al. integrated data from enzyme activity analyses and quantitative metabolite profiling into an ad hoc reduced model that included XR/XDH pathway, pentose phosphate pathway (PPP), and two lumped reactions describing glycolysis, and used this model for MCA [28].

In this work, we used the Optimization and Risk Analysis of Complex Living Entities (ORACLE) framework [29–36] to construct a population of large-scale kinetic models of glucose–xylose co-utilizing *S. cerevisiae* that includes XR/XDH pathway, glycolysis, PPP, tricarboxylic cycle (TCA), and electron transport chain (ETC). ORACLE accounts explicitly for mechanistic properties of enzymes and integrates available experimental data, network thermodynamics, and physico-chemical constraints of metabolic networks. ORACLE employs Monte Carlo sampling methods to explore the kinetic space of metabolic networks whenever information about kinetic properties is incomplete or missing, and it generates populations of models that are consistent with the experimental information. One of the steps in the ORACLE workflow involves *pruning*, where the populations of the generated models are further classified into subpopulations with distinct characteristics based on existing or follow-up experiments. The basic principles of ORACLE have been introduced in [32, 33, 37], and the method was developed and extended in [24, 29–31, 34, 36].

The main goals of this study were to: (1) analyze the impact of the network enzymes on pentose sugar utilization in a genetically recombinant XR/XDH *S. cerevisiae* strain caused by network-wide couplings and limitations rather than by a substrate competition for hexose transporters; (2) identify the sources of interdependencies between upper glycolysis and xylose uptake rate; (3) engineer genetically a recombinant *S. cerevisiae* strain with improved xylose uptake capabilities based on hypotheses generated in (2); and (4) use the experimental data acquired on the engineered strain to further improve predictive capabilities of the kinetic models.

For this purpose, we opted for a rational metabolic engineering approach involving a loop between experimental lab work and intensive computational studies. We constructed large-scale kinetic models of *S. cerevisiae* central metabolism with the XR/XDH xylose uptake pathway wherein we integrated the data collected from a recombinant *S. cerevisiae* strain growing anaerobically in a medium with a mixture of glucose and xylose. We performed an ORACLE analysis and we identified the enzymes that control the flux through XTR. However, for some of these enzymes such as HXK we could not discern with confidence whether an increase of their activities would lead to a positive or negative effect on the flux through XTR. In order to address this issue, we designed a follow-up computational pruning experiment that helped us classify models into two subpopulations of models with HXK having a positive and a negative control over XTR. We next tested experimentally how changes in hexokinase (HXK) activity would affect xylose uptake rates. The performed experiment with an *HXK2* deletion strain showed an increase in the maximum specific consumption rates of xylose. We used this result to further prune our kinetic models, and the predictions of the refined models were consistent with the cultivation data of the engineered HXK-negative recombinant *S. cerevisiae* strain.

## Results

### Cultivation and flux analysis of the glucose–xylose utilizing base strain

**Fig. 1:**
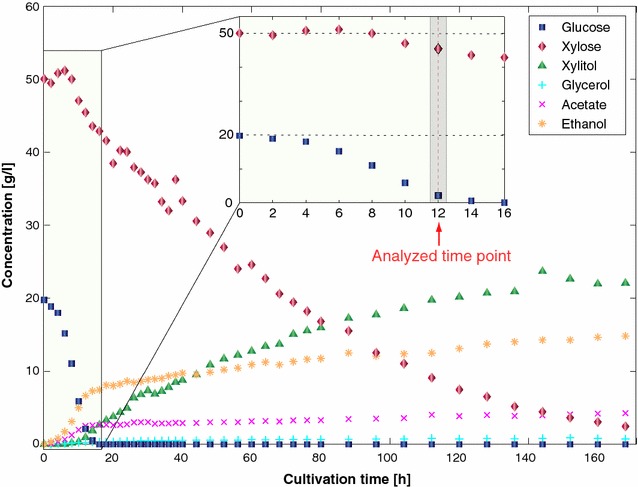
Substrates and products time evolutions of glucose–xylose co-utilization obtained for the VTT C-10880 strain growing in a bioreactor under anaerobic conditions: glucose (*blue squares*), xylose (*red diamonds*), xylitol (*green triangles*), glycerol (*cyan pluses*), acetate (*magenta crosses*), and ethanol (*orange asterisks*)

The strain VTT C-10880 was anaerobically cultivated in a batch reactor using minimal mineral medium with 20 g/l glucose and 50 g/l xylose as the main carbon source for 170 h (for more details see “Methods”). The concentrations (g/l) of glucose, xylose, xylitol, glycerol, acetate, and ethanol were measured (Fig. 1). We observed that the organism started to consume perceptible quantities of xylose between 8 and 10 h, whereas at this time period glucose was at 33% of its starting concentration. Glucose was rapidly consumed, and from *T* = 15 h onwards the yeast consumed xylose only. At *T* = 140 h, xylose was not completely consumed yet and xylitol accumulated to approximately 22 g/l.

**Fig. 2:**
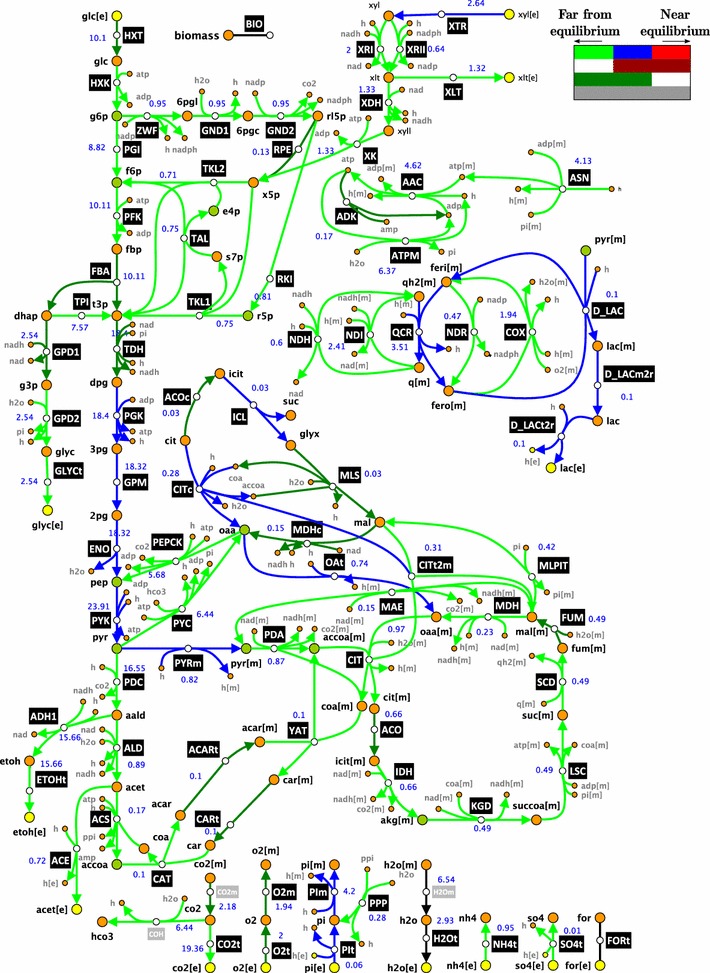
Schematic representation of the VTT C-10880 metabolism together with the displacement of the reactions from thermodynamic equilibrium. Reactions can operate (i) strictly far from thermodynamic equilibrium (*light green*), i.e., $0 < \varGamma \le 0.1$; (ii) with the middle displacements (*blue*), i.e., $0.1 \le \varGamma \le 0.9$; and (iii) strictly near equilibrium (*light red*), i.e., $0.9 \le \varGamma < 1$. Reactions whose displacements belonged to more than one of these ranges were denoted with (iv) *dark red*, for $0 < \varGamma \le 0.9$; (v) *dark green*, for $0.1 \le \varGamma < 1$; and (vi) *gray*, for $0 < \varGamma < 1$. The numerical values next to reactions denote flux values

We formulated a stoichiometric model that describes glucose–xylose co-metabolism under the studied conditions (Fig. 2; Additional file 1), and we performed the flux analysis using the specific accumulation rates calculated as the output of the kinetic model based on the cultivation data (“Methods”). We used in the flux analysis the calculated rates at time point *T* = 12 h when glucose and xylose were consumed simultaneously. We then performed thermodynamics-based flux analysis (details in “Methods”) to find a flux profile that was consistent both thermodynamically and with the available experimental data. We computed the displacement of reactions from thermodynamic equilibrium, and we found that a majority of reactions were far from equilibrium and no reactions were near equilibrium (Fig. 2; Additional file 2). This implies that for all the enzymes in the network their control over fluxes and concentrations would depend on their kinetic properties and saturation state [33, 35, 38]. We assumed that the reaction catalyzed by malic enzyme (MAE) was operating in the direction of decarboxylation because it has been observed that pyruvate carboxylase-negative strains of *S. cerevisiae* are not capable of growing on glucose indicating that MAE cannot assume the anaplerotic role of pyruvate carboxylase [39]. The upper limit on oxygen uptake rate was set to 2 mmol/gDW h for the following reasons: (1) the medium was saturated with air oxygen at the beginning of the fermentation and its concentration gradually reduced over time; we set the upper bound on oxygen utilization rate to reflect the possibility that there was a residual oxygen activity at the analyzed time point; (2) molecular oxygen appears as a substrate of the used biomass reaction (Additional files 1, 3); at the analyzed time point, the organism was still growing and the model required a small amount of oxygen to describe the growth.

### Factors affecting xylose uptake during glucose–xylose co-metabolism

It has been proposed that the reason for slow consumption of xylose in the beginning of the glucose–xylose co-utilization phase is due to substrate competition for high-affinity hexose transporters [40]. In this study, we investigated if the other factors that emerge from the properties of the metabolic network and that are unrelated to the competition for transporters could also impact the xylose uptake. For example, it has been discussed in the literature that availability of the cofactors NAD+ and NADPH in the metabolic network can be one of the limiting factors in xylose utilization [12].

**Fig. 3:**
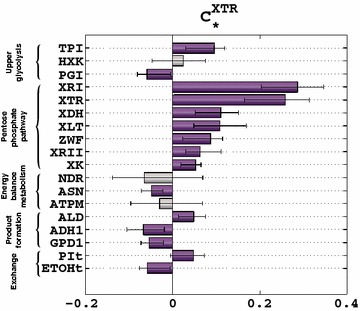
Control coefficients of the xylose uptake during glucose–xylose co-utilization. The *bars* represent the mean values of the control coefficients through xylose transporters (XTR). The *error bars* denote the 1st and the 3rd quartile of the control coefficients with respect to their mean value, i.e., 50% of the samples closest to the mean value are within the *error bars*. The enzymes whose distributions of control coefficients were spread around zero, i.e., whose values did not allow us to predict with certainty the responses of metabolic network, are marked in *gray*

We used the ORACLE methodology (“Methods”) to build a population of consistent models. More precisely, we generated ~467,000 system-wide profiles of flux and concentration control coefficients.^1^ We then focused on the flux control coefficients of the xylose uptake (XTR) and identified the top 18 enzymes with respect to the absolute value of their control coefficients (Fig. 3). Among the 18 enzymes, 7 were from the pentose phosphate pathway (ZWF, XTR, XRI, XRII, XDH, XK, and XLT), 3 were from the upper glycolysis (HXK, PGI, and TPI), 3 were related to energy balance through oxidative phosphorylation and its consumption through maintenance (ASN, NDR, and ATPM), 3 were enzymes controlling the formation of ethanol (ADH1), acetate (ALD), and glycerol-3-phosphate (GPD1), and 2 were enzymes catalyzing exchanges with the extracellular environment (ETOHt and PIt). Downstream enzymes relating to lower glycolysis, citric acid cycle, and glyoxylate shunt were found to have little to almost no control over xylose uptake (Additional file 4).

The positive control over the xylose uptake was distributed over ten enzymes, i.e., ZWF, XTR, XRI, XRII, XDH, XK, XLT, TPI, ALD, and PIt, whereas 5 enzymes, i.e., PGI, ASN, ADH1, GPD1, and ETOHt, had negative control (Fig. 3). XRI had the highest positive control over XTR with $C_{\text{XRI}}^{\text{XTR}}$ ~ 0.29 indicating that a twofold increase (100%) of XRI activity would result in a 1.29-fold increase (29%) in the xylose uptake rate. ADH1 had the highest negative control over this rate with $C_{\text{ADH1}}^{\text{XTR}}$ ~ −0.07 indicating that a twofold increase of ADH1 activity would result in an ~7% decrease in the xylose uptake rate.

For three enzymes, i.e., HXK, ATPM, and NDR, the distributions of the control coefficients, $C_{\text{HXK}}^{\text{XTR}}$, $C_{\text{ATPM}}^{\text{XTR}}$, and $C_{\text{NDR}}^{\text{XTR}}$, were spread around the zero value (Fig. 3), and with the available data we were unable to predict with certainty if the manipulation of these enzymes would affect the xylose uptake rate in a positive or negative manner. This implied that we might have two distinct subpopulations of models with one population predicting a negative control of HXK over XTR ($C_{\text{HXK}}^{\text{XTR}} < 0)$, and the other predicting a positive control ($C_{HXK}^{XTR} > 0$).

In addition, we observed a rather strong negative correlation between the values of $C_{\text{HXK}}^{\text{XTR}}$ and the values of both $C_{ATPM}^{XTR}$ and $C_{\text{NDR}}^{\text{XTR}}$. We computed the correlation between these control coefficients and the Pearson coefficient for the correlation of $C_{\text{HXK}}^{\text{XTR}}$ and $C_{\text{ATPM}}^{\text{XTR}}$ was −0.68, the one of $C_{\text{HXK}}^{\text{XTR}}$ and $C_{\text{NDR}}^{\text{XTR}}$ was −0.67, whereas the one for the correlation of $C_{\text{ATPM}}^{\text{XTR}}$ and $C_{\text{NDR}}^{\text{XTR}}$ was +0.76.

### Factors affecting ATP consumption and generation

Interestingly, the enzymes participating in the generation and consumption of ATP appeared to have considerable control over xylose uptake, even though they have been traditionally overlooked when the system was studied from the perspective of carbon flow.

**Fig. 4:**
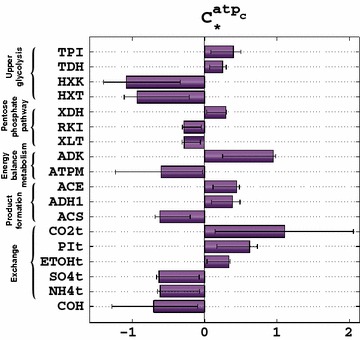
Control coefficients of the cytosolic ATP concentration during glucose–xylose co-utilization. The *bars* represent the mean values of the control coefficients, and the *error bars* denote the 1st and the 3rd quartile of the control coefficients with respect to their mean value, i.e., 50% of the samples closest to the mean value are within the *error bars*

Therefore, we further investigated how changes in the activities of the enzymes would affect the levels of cytosolic ATP (Fig. 4). We used the population of ~467,000 kinetic models to identify the top 16 enzymes controlling the levels of this metabolite. Out of the top 16 enzymes, three were from the upper glycolysis (HXT, HXK, and TPI), two were from the pentose phosphate pathway (RKI and XDH), two were directly related to ATP consumption and production (ADK and ATPM), two were from acetate metabolism (ACS and ACE), five were exchange reactions with the extracellular environment (ETOHt, NH_4_t, SO_4_t, PIt, and CO_2_t), and one was COH which is catalyzing the reversible conversion of carbonic acid to CO_2_ and water. HXK had the primary negative control on the levels of ATP with $C_{\text{HXK}}^{{{\text{atp}}_{c} }}$ ~ −1.07 indicating an important increase in the levels of cytosolic ATP upon reduction in the activity or deletion of *HXK2.* HXT also had a strong negative control over the levels of cytosolic ATP ($C_{\text{HXT}}^{{{\text{atp}}_{c} }}$ ~ −0.93). The strongest positive control over this metabolite was surprisingly in CO_2_t ($C_{{{\text{CO}}_{2} {\text{t}}}}^{{{\text{atp}}_{c} }}$ ~ 1.1) and in ADK ($C_{\text{ADK}}^{{{\text{atp}}_{c} }}$ ~ 0.95).

Considering that ATP impacts a large number of reactions in the network including HXK and that HXK had a strong control over ATP implied that there was a strong coupling through ATP in the network. This together with the hypothesis that an increase in the activity of HXK would decrease the xylose uptake rate was intriguing, and we decided to verify this hypothesis by performing an experiment on a strain with attenuated HXK activity.

### *HXK2* deletion improves xylose uptake

**Fig. 5:**
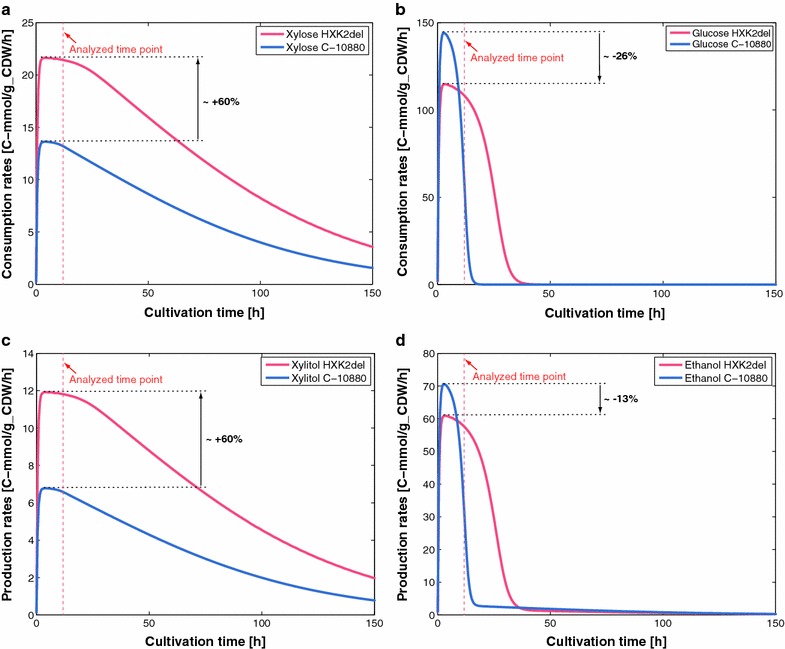
Fermentation profiles of the C-10880 strain (*blue*) and of the engineered *HXK2*-deficient strain (*red*): specific xylose consumption rates (**a**), specific glucose consumption rates (**b**), specific xylitol production rates (**c**), and specific ethanol production rates (**d**)

*Saccharomyces cerevisiae* has three enzymes phosphorylating glucose, HXK1, HXK2, and GLK1, of which HXK2 is the predominant enzyme under consumption of glucose. *HXK2* is expressed when growing on glucose, but when growing on non-fermentable carbon sources *HXK2* expression is repressed and the expression of *HXK1* and *GLK1* de-repressed [41]. As HXK2 contributes to the predominant hexokinase activity on glucose and is the main enzyme conveying catabolite repression of these two hexokinases, we constructed a recombinant *S. cerevisiae HXK2*-deficient strain (see Methods). The engineered strain indeed displayed a clear improvement of the xylose uptake rate with values of specific rate of xylose consumption consistently higher than the ones of the base strain with the maximum values showing improvement by approximately 60% (Fig. 5a). The increase in the xylose consumption led also to an increase in the production of xylitol with a 60% increase in the maximum specific xylitol production rate (Fig. 5c). Conversely, deletion of the *HXK2* gene resulted in (a) reduced maximal glucose specific consumption rate by 26% with the depletion of glucose occurring at approximately *T* = 36 h compared to *T* = 16 h of the base strain (Fig. 5b); and (b) reduction of the maximal specific accumulation rate of ethanol by 13% (Fig. 5d).

**Fig. 6:**
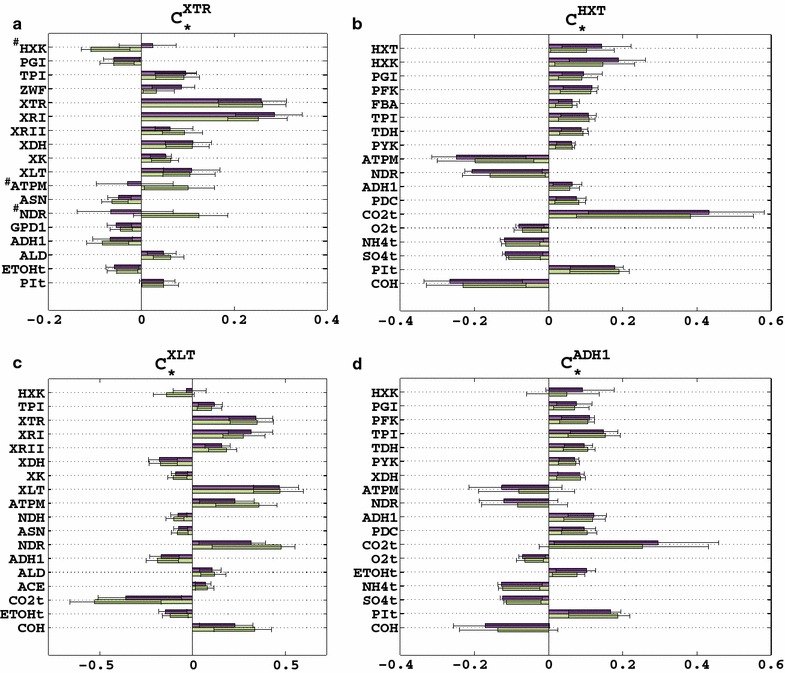
Control coefficients of the original (*purple*) and refined (*green*) models for xylose uptake rate, XTR (**a**), glucose uptake rate, HXT (**b**), xylitol production, XLT (**c**), and alcohol dehydrogenase, ADH1 (**d**). The *bars* represent the mean values of the control coefficients, and the *error bars* denote the 1st and the 3rd quartile of the control coefficients with respect to their mean value, i.e., 50% of the samples closest to the mean value are within the *error bars*. With # are denoted the enzymes whose distributions of control coefficients were spread around zero in the original (*purple*) models

The computed control coefficients for specific glucose uptake, $C_{\text{HXK}}^{\text{HXT}}$, and for specific productivity of ethanol, $C_{\text{HXK}}^{\text{ADH1}}$, were in qualitative agreement with the experiments, i.e., the trends of the expected metabolic responses and the relative magnitudes of these control coefficients were in agreement with the experiments. The computed mean values of $C_{\text{HXK}}^{\text{HXT}}$ and $C_{\text{HXK}}^{\text{ADH1}}$ were, respectively, 0.18 and 0.095 (Fig. 6b, d), i.e., a twofold decrease in the activity of HXK would lead to 9 and 4.75% decrease in the corresponding specific productivities.

### Pruning the models

Based on the experimentally obtained information, we rejected all sampled models with $C_{\text{HXK}}^{\text{XTR}} > 0$ and ended up with a population of ~204,000 models. The mean value of $C_{\text{HXK}}^{\text{XTR}}$ shifted to a value of −0.11 after the pruning compared to 0.02 before the pruning (Fig. 6a). The value of −0.11 indicated that a twofold increase in the activity of HXK would result in the reduction of the specific xylose uptake by 11%.

Interestingly, the pruning based on $C_{\text{HXK}}^{\text{XTR}}$ did not have a significant effect on the distribution of most of the other control coefficients with the exception of $C_{\text{ATPM}}^{\text{XTR}}$ and $C_{\text{NDR}}^{\text{XTR}}$. Before the pruning, these two control coefficients had distributions spread around the zero value with the means of −0.02 and −0.05, respectively (Fig. 6a). After the pruning, the distributions of both $C_{\text{ATPM}}^{\text{XTR}}$ and $C_{\text{NDR}}^{\text{XTR}}$ were shifted toward positive values with their respective means of 0.10 and 0.13. The pruning results suggested that the negative correlation between the values of $C_{\text{HXK}}^{\text{XTR}}$ and the values of both $C_{\text{ATPM}}^{\text{XTR}}$ and $C_{\text{NDR}}^{\text{XTR}}$ that we discovered on the whole population of 467,000 models still existed on the pruned population. We computed the correlations for the pruned population of control coefficients and the Pearson coefficient for the correlation of $C_{\text{HXK}}^{\text{XTR}}$ and $C_{\text{ATPM}}^{\text{XTR}}$ was −0.56, the one of $C_{\text{HXK}}^{\text{XTR}}$ and $C_{\text{NDR}}^{\text{XTR}}$ was −0.58, whereas the one for the correlation of $C_{\text{ATPM}}^{\text{XTR}}$ and $C_{\text{NDR}}^{\text{XTR}}$ was +0.73.

In silico predictions of the pruned control coefficients were consistent with all the experimentally observed effects of *HXK2* deletion on the behavior of the engineered strain. More precisely, in qualitative agreement with the performed experiments the refined population of control coefficients predicted that *HXK2* deletion would (a) decrease the glucose uptake, i.e., the mean of computed $C_{\text{HXK}}^{\text{HXT}}$ was ~$0.14$ (Fig. 6b); (b) reduce the ethanol production, i.e., the mean $C_{\text{HXK}}^{\text{ADH1}}$ was ~0.05 (Fig. 6d); and (c) increase the xylitol production, i.e., the mean $C_{\text{HXK}}^{\text{XLT}}$ was ~−0.14 (Fig. 6c). The refined models also consistently predicted that the *HXK2* deletion would increase the flux through xylulose kinase (XK) with the mean $C_{\text{HXK}}^{\text{XK}}$ of ~−0.08 (Additional file 5). Indeed, computed as the difference between the experimental values for xylose consumption and xylitol production fluxes (Fig. 4a, c), the estimated flux through XK was higher in the *HXK2* deletion strain than in the reference strain.

### Network-wide effects of altered HXK activity

**Fig. 7:**
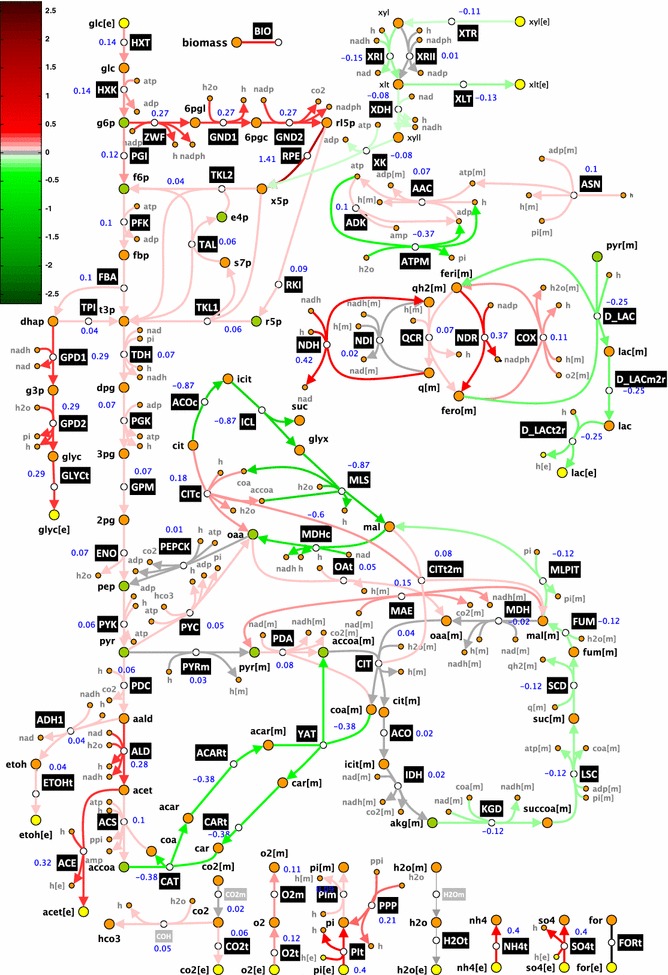
Predicted effects of HXK manipulations on the metabolic fluxes. An increased activity of HXK would result in reactions with: increased flux (*red*), decreased flux (*green*), or negligible changes in flux (*gray*). Higher intensity of *red* or *green* indicates larger changes in fluxes. The numerical values shown above reactions denote the mean control coefficients upon changes in HXK, i.e., $C_{\text{HXK}}^{*}$ where *asterisk* denotes any metabolic flux in the network

Metabolic control analysis indicated that *HXK2* deletion would cause significant rewiring of the metabolic network. Reactions from the glycolysis, pentose phosphate pathway with the exception of xylose uptake pathway, oxidative phosphorylation with the exception of lactate-related reactions, glycerol metabolism, acetate and ethanol production, and exchange with the extracellular environment would reduce their fluxes compared to the parental strain (Fig. 7; Additional file 4). For instance, a twofold decrease of the activity of HXK would reduce the fluxes in the glycolysis from 5.6% (PYK) to 14% (HXK) and the fluxes in the pentose phosphate pathway from 4% (TKL2) to 141% (RPE). In contrast, decreasing the activity of HXK would result in an increased activity of the xylose uptake pathway, glyoxylate shunt, tricarboxylic acid cycle, and ATPM which manages a series of energy-requiring events inside the cell without leading to net formation of biomass including futile cycles and maintenance of the proton gradient and electrical potential (Fig. 7). A twofold decrease of activity of HXK would increase fluxes through XTR (by 5%), XRI (by 7%), XLT (by 6.6%), XDH (by 4%), and XK (by 4%) in the xylose uptake pathway, fluxes through MDHc (by 30%) and ICL, MLS, and ACOc in the glyoxylate shunt (by 43%), fluxes through KGD, LSC, SCD, and FUM in the TCA cycle (by 6%), and ATPM (by 18%).

Furthermore, a twofold reduction in the HXK activity would reduce ATP turnover (by ~3.2%) where all ATP-producing reactions (PGK, PYK, and AAC) and most of ATP-consuming reactions (HXK, PFK, PYC, ADK, ACS, PEPCK, and biomass) would have reduced fluxes (Fig. 7; Additional file 4). The only exceptions would be XK and ATPM, whose fluxes would increase (by 4.2 and 18%, respectively).

### Network-wide correlations of HXK, NDR, and ATPM

Interestingly enough, an increased activity of NDR and ATPM, whose control over XTR negatively correlated with the one of HXK (“Results”), would have similar effects on the metabolic network as the *HXK2* deletion (Additional files 4, 6, 7). Notable differences in the metabolic responses would be in (1) a reduced flux through XK and XDH (by 15%) with a twofold ATPM increase, and by 21% with a twofold increase of NDR activity; this implies that metabolic engineering strategies involving NDR and ATPM would improve xylose uptake but the carbon taken up would be used almost exclusively for xylitol production; (2) reduction in the TCA fluxes (ACO, IDH, KGD, LSC, SCD, FUM, and MDH) from 33 to 94%, and reduced ATPM by 11%, with a twofold increase of the NDR activity. The twofold increase in ATPM and in NDR activity would also result in reduced turnover of cytosolic ATP by 9 and 17%, respectively (versus 3.2% of the one in the case of HXK) and reduced turnovers of cytosolic NADH (by 11% for ATPM and 14% for NDR versus 3.4% for HXK) and NADPH (by 41% for ATPM and 31% for NDR versus 15% for HXK).

The negative correlation between HXK and ATPM was expected as the reduced HXK activity results in reduced glycolytic fluxes and as a consequence in reduced ATP production and availability. A similar effect on ATP can be reproduced by increasing non-growth-associated ATP maintenance (ATPM). In contrast, the correlation between NDR and both HXK and ATPM was more surprising. Our analysis revealed that this correlation is due to the stoichiometric coupling between NDR and the growth in the analyzed physiological condition (Additional file 8). Specifically, we performed the flux variability analysis (FVA), sampled the space of steady-state fluxes, and found that the growth is perfectly coupled with NDR. For the smallest feasible values of the flux through NDR (0.016 mmol/gDW/h), the growth was small (0.002 1/h). In contrast, for the highest feasible values of NDR flux (0.48 mmol/gDW/h), the growth increased almost 100-fold to 0.19 1/h (Additional file 8). The growth increase resulted in an increase of the growth-associated ATP requirements, and the maximal feasible flux value of ATPM for low growth of 18.28 mmol/gDW/h reduced to the one of 12.55 mmol/gDW/h for high growth.

## Discussion

*HXK2* is the gene that has a main role in glucose phosphorylation, and it could be expected for a strain that lacks this gene to exhibit decreased glucose consumption levels. In the performed experiment, *HXK2* deletion decreased the maximal glucose consumption rate by 26% compared to the parental strain. However, for predicting the responses of the other fluxes in the network like xylose uptake rate, a systems-oriented tool such as MCA is needed. *HXK2* deletion would have been an unlikely target for improving xylose consumption without the MCA analysis. Indeed, the pruned MCA models successfully predicted an increase in xylose consumption and xylitol production and a decrease in glucose consumption and ethanol production of the *HXK2-*deficient strain.

Considering that we modeled the transporters of glucose and xylose as enzymes without competitive inhibition and that we neglected the HXK2 catabolic repression, MCA results implied that there are factors other than competition for the transporters or glucose repression that could negatively affect the xylose uptake. MCA suggested that decreasing HXK activity would increase the concentration of cytosolic ATP (Fig. 4) and the rate of xylulose kinase (XK) (Additional file 5), thus allowing improved xylose uptake (Fig. 7).

Previous experimental studies investigated some of the enzymes identified here for improved xylose uptake rate (Fig. 3). Jeppsson et al. [22] reported that a reduced flux through the oxidative branch of pentose phosphate pathway (PPP) causes reduced xylose uptake rate in a xylose-only fed recombinant *S. cerevisiae* strain. Consistent with [22] where the ZWF1 gene was deleted to reduce the NADPH-producing flux, our predictions indicate that lowering the activity of glucose-6-phosphate-1-dehydrogenase (ZWF) would result in reduced xylose uptake rate. Our predictions are also in agreement with two experimental studies regarding the effects of xylose reductases (XRI and XRII) [18] and xylitol dehydrogenase (XDH) [21] on xylose uptake rate.

Our predictions additionally suggest that increasing the PGI activity would result in a reduced xylose uptake rate. During glucose–xylose fermentations studied here, PGI converts glucose 6-phosphate to fructose 6-phosphate, and therefore a higher PGI activity results in a reduced flux through the oxidative branch of PPP. This is consistent with the experimental study [22], since in batch fermentations with xylose as the sole carbon source performed in [22] PGI converts fructose 6-phosphate to glucose 6-phosphate, and a lower PGI activity reduces the flux through the oxidative branch of PPP.

Toivari et al. [20] obtained improved xylose uptake with a CEN.PK2 strain with an increased xylulose kinase (XK) activity both in xylose-only and glucose–xylose anaerobic batch fermentations. These experimentally observed effects of *XKS1* overexpression are in agreement with the quantitative predictions (Fig. 3). In contrast, *XKS1* overexpression in a different strain background (including overexpressed non-oxidative PPP genes, *gre3* deletion, and *xylB* originating from *Escherichia coli*) and in an anaerobic xylose-only batch fermentation resulted in slightly reduced xylose uptake [27]. These findings are in line with the results from other studies on XK role in xylose uptake for *S. cerevisiae* which indicate that only a fine-tuned XK overexpression leads to improved xylose fermentation [42–45].

Furthermore, in a recent study, Trausinger et al. [28] performed MCA of xylose assimilation of *S. cerevisiae* strain grown under anaerobic conditions on xylose as a sole carbon source. The computed flux control coefficients for xylose uptake with respect to XR, XDH, XK, and ZWF in [28] are consistent with the ones found in the current study.

While the refined models successfully predicted the trends of the metabolic responses of the *HXK2* deletion, the magnitudes of the responses predicted by the refined control coefficients did not accurately match the experimentally observed ones. These discrepancies can originate from any of the following reasons or their combination: (a) control coefficients represent a log-linear approximation of the metabolic responses upon *small* changes around the studied steady state in an enzyme activity, and they typically provide a good ranking of enzymes that control the studied fluxes or concentrations. In this study, we performed a gene deletion that is a major perturbation of a metabolic network; (b) it has been shown that in a *HXK2-*deficient *S. cerevisiae* strain the transporters *HXT2, HXT4, HXT6, HXT7, HXT8*, and *HXT16* have higher gene expression levels [46]. Of these hexose transporters, especially the high-affinity transporters *HXT2*, *HXT4*, and *HXT7* are active in xylose transport [14, 47]. The absence of *HXK2* repressive effect on the hexose transporters involved in the transport of xylose might be the reason for the higher than predicted consumption rate of xylose in the *HXK2*-deficient strain; and (c) the population of kinetic models was computed for the time point *T* = 12 h when glucose and xylose were co-utilized, whereas the maximal consumption and production rates compared in two strains were measured at *T* = 3.5 h when glucose was predominantly utilized.

Finally, although the estimated values for specific rates of xylose and glucose conversion and ethanol and xylitol production may be affected by variations in experimental measurements and by a bias in data smoothing, there was a clear and significant improvement in specific xylose conversion rate upon *HXK2* deletion and it was predicted by the model. ORACLE is designed to handle the uncertainty in data, and the populations of sampled models capture the trends in the metabolic responses upon *HXK2* deletion.

## Conclusions

We described here a design–build–test cycle including the steps of modeling, identifying targets for engineering, testing the gene modification in a engineered strain, and further refining the models. More specifically, we used MCA to explore potential improvements of xylose utilization in glucose–xylose co-fermenting *S. cerevisiae*. From this analysis, we postulated a hypothesis that the HXK enzyme has negative control over specific xylose uptake rate and we performed an experiment to verify it. Although the effects of *HXK2* deletions on glucose repression have been studied before [48–50], a deletion of *HXK2* has not been studied as a means to improve xylose consumption in glucose–xylose co-fermenting *S. cerevisiae* strains. The influence of carbon catabolite repression on xylose metabolism in *S. cerevisiae* strains through deletion of *MIG1* and *MIG2* genes was studied previously; however, no improvement of xylose consumption during batch cultivations was observed [51].

The performed in silico analysis demonstrated that modifying HXK activity would substantially rebalance the network fluxes, and that these effects strongly correlate with the ones of NDR and ATPM. We discovered that the correlation of effects of HXK, ATPM, and NDR on the whole network is an emerging property of the stoichiometric coupling through ATP.

We engineered an *HXK2-*deficient strain and the cultivation results validated the postulated hypothesis. We used the experimental information to prune the population of our kinetic models and the refined models were in qualitative agreement not only with the response of XTR to *HXK2* deletion but also with the responses of HXT, ADH1, XLT, and XK. Pruning is one of the important features of ORACLE as it allows a straightforward refinement of models based on experimental information about the metabolic responses to gene modifications and it improves the efficiency of the design–build–test cycle. This is an advantage with respect to the conventional parameter estimation methods where the integration of this kind of information is challenging.

The present study aims to improve the xylose uptake rate during a mixed glucose–xylose co-utilization and to elucidate a complex interplay between upper glycolysis and xylose uptake. It is a step toward obtaining strains with economically viable yields and specific productivity of ethanol from both glucose and xylose as carbon sources. The next step in this direction is to identify targets that will allow for recovered glucose uptake and ethanol specific productivity while retaining the improved xylose uptake of the *HXK2* deletion strain. Our models suggest that one of such targets might be TPI which can also be used together with XDH to improve the yield of ethanol from both glucose and xylose.

## Methods

### Strains and plasmids

The yeast strain used in the study was VTT C-10880, i.e., [CEN.PK113-1A (*URA3. HIS3. LEU2. TRP1. MAL2*-*8* ^*c*^ *.SUC2), ura3::XYL1 XYL2, xks1::XKS1*]. *XYL1* and *XYL2* genes of *S. stipitis* were chromosomally integrated into *URA3* locus under *PGK1* and *ADH1* promoters, respectively. The integration cassette was constructed as described earlier [20]. *XKS1* of *S. cerevisiae* was integrated into *XKS1* locus as described below.

The *XKS1* expression cassette p*ADH1*m-*XKS1*-t*ADH1* was released as a *Bam*HI fragment from plasmid pMLV14 (BluescribeM13) and ligated into the bacterial plasmid pMLV24. The plasmid pMLV24 contains *loxP*-*S. cerevisiae MEL5* (encoding α-galactosidase)-*loxP* marker cassette with 60-bp flanking regions for targeting to the *XKS1* locus. The *XKS1* flanking regions were from nucleotides −250 to −192 and from nucleotides 1801 to 1861, where numbers are relative to the nucleotide A in the *XKS1* ATG start codon. The *Bam*HI cloning site was included in the *XKS1* 5′ flanking sequence. The resulting plasmid, containing *XKS1*, was named pMLV46. The expression cassette for *XKS1*, together with the *loxP*-*MEL5*-*loxP* marker, was released from pMLV46 with *Pst*I and *Spe*I and introduced into yeast cells by transformation. Blue-colored, *MEL5* (α-galactosidase)-expressing yeast colonies were collected from agar-solidified YP containing 2% w/v d-galactose, supplemented with 5-bromo-4-chloro-3-indolyl-α-d-galactopyranoside (*X*-*α*-*Gal*, 40 μg/ml). To remove the *MEL5* marker cassette from the yeast chromosome, the transformant was retransformed with a plasmid pSH47 [52], expressing the Cre recombinase. Integration of the expression cassette p*ADH1*m-*XKS1* **-**t*ADH1* into the *XKS1* locus in the genome was verified with PCR. The yeast strain generated was named VTT C-10880.

Plasmid pKK27-1 was constructed by releasing KanMX4 from pFA6a-KanMX4 [53] by *Bam*HI + *Eco*RI digestion. KanMX4 was further ligated into YEplac195 [54] digested with *Bam*HI + *Eco*RI resulting in pKK27-1.

*HXK2* of VTT C-10880 was deleted by integration of KanMX4 into *HXK2* locus. KanMX4 deletion cassette was amplified by PCR from pKK27-1 plasmid using forward 5′ TCTGTTTAGCTTGCCTCGTC and reverse 5′ CACTGGATGGCGGCGTTAGTA primers with the homologous flanking sequences to *HXK2* locus (−27 to −33 for the forward primer and +1 to +60 for the reverse primer). The resulting PCR product was integrated into the genome of VTT C-10880 by homologous recombination by following the transformation procedure of Woods and Gietz [55]. Transformants were selected on YPD medium containing 200 µg/ml G418. The deletion of *HXK2* was verified by PCR using primer pairs 5′GGTTGTAGGAATATAATTCTC3′ and 5′CAACGCTACCTTTGCCATGT3′ and 5′ATCCTGATATGAATAAATTG3′ and 5′CATGTTCACATAAGTAGAAAAAGGGCACC3′.

### Bioreactor cultivations

The strain VTT C-10880 and the *HXK2* deletion strain were studied for 170 h in 1.5-l batch cultures in Biostat B bioreactor (Sartorius, Göttingen, Germany) at pH 5 and 30 °C using agitation at 200 rpm, under anaerobic conditions with 0.5 l/min nitrogen flushed to reactor headspace. The *HXK2* deletion strain was cultivated in duplicate experiments. The inocula were prepared by transferring the cells from an YPD plate to 25 ml mineral medium in 100-ml Erlenmeyer flasks and incubated on a plate shaker (150 rpm, 30 °C) overnight. The inocula were further transferred into 75 ml mineral medium in 250-ml Erlenmeyer flasks and incubated (150 rpm, 30 °C) for 4-6 h. The inocula were centrifuged (2000 rpm, 4 °C, 5 min) and the cells were resuspended into the growth medium lacking the source of carbon. Initial cell dry weight in bioreactor was 0.15 g/l. The minimal mineral medium [56] contained 20 g glucose/l, 50 g xylose/l, 22 mg uracil/l, 5 g (NH_4_)_2_SO_4_/l, 3 g KH_2_PO_4_/l, 0.5 g MgSO_4_·7H_2_O/l, 15 mg C_10_H_14_N_2_Na_2_O_8_·2H_2_O (EDTA)/l, 4.5 mg ZnSO_4_·7H_2_O/l, 0.84 mg MnCl_2_·2H_2_O/l, 0.3 mg CoCl_2_·6H_2_O/l, 0.3 mg CuSO_4_·5H_2_O/l, 0.4 mg Na_2_MoO_4_·2H_2_O/l, 4.5 mg Na_2_MoO_4_·2H_2_O/l, 3.0 mg FeSO_4_·7H_2_O/l, 1.0 mg H_3_BO_3_/l, 0.1 mg KI/l, 0.05 mg biotin/l, 1.0 mg Ca-Pantothenate/l, 5 mg nicotinic acid/l, 25 mg myo-inositol/l, 1.0 mg thiamine HCl/l, 1.0 mg pyridoxol HCl/l, and 0.2 mg *p*-aminobenzoic acid/l.

### Sampling and analyses

Samples were taken frequently with a sampling robot (Medicel, Helsinki, Finland) into a cold bath where the samples froze at the temperature of −30 °C. The frozen samples were thawed, OD600 and cell dry weight (CDW) measurements were performed, and supernatants were collected for HPLC and CE measurements. Concentrations of xylose, glucose, glycerol, xylitol, ethanol, and acetate were measured with a Waters Alliance 2690 HPLC system with a Waters 2410 RI detector (Waters Corporation, Milford, USA). Aminex HPX-87H column and Aminex fast acid analysis column (BioRad, USA) were used in series with 5 mM H_2_SO_4_ (Merck Titrisol) with a flow rate of 0.6 ml/min to separate the analytes at 55 °C. Concentrations of lactic acid, succinic acid, and malic acid were measured using a P/ACE MDQ capillary electrophoresis system (Beckmann Coulter Inc., Fullerton, USA) equipped with a PDA detector [57]. Concentrations of CO_2_, O_2_, and ethanol in the exhaust gas of the bioreactor were measured using an Innova gas analyzer (Innova Air Tech Instruments A/S, Ballerup, Denmark).

### Quantitative analysis of fermentation measurements

A kinetic model was applied to smooth the measurement data to reduce the noise for better estimation of the calculated continuous variables such as specific accumulation rates. Performing these calculations directly from the measured data would provide data too noisy for interpretations. The dynamic system of anaerobic yeast cultivation was described by a set of non-linear ordinary differential equations (ODEs), with the system components comprising concentrations of biomass, the substrates glucose and xylose, the products ethanol, glycerol, xylitol, acetate, lactate, succinate, and malate, and the off-gas CO_2_.

The consumption of both substrates, glucose and xylose, was described by Monod kinetics with an initial lag phase. Concentrations are written as *c*, the subscript *s* stands for either of the substrates, glucose and xylose, respectively, and the subscript *x* for biomass. The maximum specific substrate uptake rate is denoted by $Y_{s}$, and the monod dynamics are characterized by the parameter $k_{m,s}$. The lag phase is determined by the time constant $\tau$.

$$ \frac{{{\text{d}}c_{s} }}{{{\text{d}}t}} = - Y_{s}\;c_{x}  \frac{{c_{s} }}{{k_{m,\;s} + c_{s} }} \left( {1 - e^{{ - \frac{t}{\tau }}} } \right) $$

Growth of the biomass is determined by the rate of substrate consumption, and thus

$$ \frac{{{\text{d}}c_{x} }}{{{\text{d}}t}} = -  \mu_{\text{glu}} \frac{{{\text{d}}c_{\text{glu}} }}{{{\text{d}}t}} -  \mu_{\text{xyl}} \frac{{{\text{d}}c_{\text{xyl}} }}{{{\text{d}}t}} $$

with the specific maximum growth rates $\mu_{\text{glu}}$ and $\mu_{\text{xyl}}$ on glucose and xylose, respectively.

The products, i.e., ethanol, xylitol, glycerol, acetate, and minor acids, as well as the off-gas CO_2_, denoted by the subscript *p*, are released proportionally to substrate consumption with a yield parameter $Y_{s,p}$ specific to the substrate and the respective product.

$$ \frac{{{\text{d}}c_{p} }}{{{\text{d}}t}} = -  Y_{{{\text{glu}},\;p}} \frac{{{\text{d}}c_{glu} }}{{{\text{d}}t}} -  Y_{{{\text{xyl}},\;p}} \frac{{{\text{d}}c_{\text{xyl}} }}{{{\text{d}}t}} $$

Ethanol is evaporating from the broth during the cultivation in non-negligible amounts. In order to fit the model to the measurement data, ethanol evaporation is included in the model by first-order dynamics. The measured ethanol concentration is then described by

$$ \frac{{{\text{d}}c_{{{\text{eth,}}\;{\text{mess}}}} }}{{{\text{d}}t}} = \frac{{{\text{d}}c_{\text{eth}} }}{{{\text{d}}t}} - e c_{\text{eth}} $$

with the evaporation parameter *e* being a linear function of the fermentation broth volume *v*

$$ e = a - b v. $$

The parameters of the model were fit to the available measurement data by means of a maximum likelihood estimator in two subsequent steps. In a first step, the parameters for the coupled system containing the ODEs for glucose, xylose, and biomass concentrations were identified, and in a following step all the remaining parameters were estimated (Additional file 9). Parameter estimations and simulations were performed with Matlab, version 7.10.0 (R2010a) (Mathworks, Natick, MA, USA).

### Mathematical models of xylose–glucose utilizing metabolic network

#### Stoichiometric model

The model of the *S. cerevisiae* recombinant xylose–glucose co-utilizing metabolic network consists of 102 reactions and 96 intracellular metabolites distributed over cytosolic and mitochondrial compartment and extracellular environment (Fig. 2). The model contains XR/XDH xylose assimilation pathway [12]. The metabolites have been categorized as cytosolic or mitochondrial according to the physiological information on their cellular compartmentalization. All reactions in the model are completely balanced, i.e., reactions are atomically balanced with respect to carbon, protons, nitrogen, phosphorus, etc.

Although a family of hexose transporters, Hxt1p–Hxt17p, Gal2p, and Snf3p [40, 58, 59], facilitates the xylose assimilation in *S. cerevisiae*, in this study the xylose and glucose transport were modeled as independent reactions catalyzed by separate enzymes. This way, we are able to investigate the relations between upper glycolysis and xylose uptake pathway that are not caused by the competition for transporters of these two pathways.

#### Kinetic model

For each of the reactions within the studied metabolic network, a kinetic mechanism has been assigned based on the available literature data. The kinetic mechanisms used include reversible Michaelis–Menten kinetics, ordered Bi–Bi, Bi–Ter, and Ter–Bi [60]. Whenever the exact kinetic mechanism was not known, generalized approximations of enzymatic mechanisms such as generalized reversible Hill [61] or convenience kinetics [62] have been used. The kinetic model includes also a model of allosteric regulation for the phosphofructokinase (PFK) reaction. The mechanism for this reaction is modeled as Hill kinetics with the Hill coefficient *h* = 4, where adenosine monophosphate (AMP), acting as an external activator, and adenosine triphosphate (ATP), acting as an inhibitor, bind to the same site. Detailed account about the used kinetic mechanisms is given in the Additional files 3 and 10.

### Displacement from thermodynamic equilibrium

For any metabolic reaction, the displacement from the thermodynamic equilibrium can be expressed as a function of the equilibrium constant, $K_{\text{eq}}$, defined as the ratio between the products of the forward and backward rate constants, and the concentration of the involved substrates, $S_{i}$, and products, $P_{j}$, i.e.

$$ \varGamma = \frac{1}{{K_{\text{eq}} }}\frac{{\mathop \prod \nolimits P_{j} }}{{\mathop \prod \nolimits S_{i} }}. $$

The equilibrium displacement, $\varGamma$, is related to the Gibbs free energy difference, $\Delta G$, through the following thermodynamic equation [38, 63]:

$$ \Delta G = {\text{RT}} \ln \varGamma $$

with $R$ being the ideal gas constant and $T$ the standard temperature. We assume that the reactions produce spontaneously the products $P_{j}$, i.e., $\Delta G < 0$ and consequently $\varGamma < 1$. When a reaction gets close to its thermodynamic equilibrium, $\varGamma$ is approaching 1. At equilibrium, $\varGamma = 1$ and

$$ \left. {\frac{{\mathop \prod \nolimits P_{j} }}{{\mathop \prod \nolimits S_{i} }}} \right|_{\text{eq}} = - {\text{RT}} \ln K_{\text{eq}} = \Delta G^\circ , $$

where $\Delta G^\circ$ denotes the standard Gibbs energy of reaction.

### Efficient building of large-scale kinetic models

**Fig. 8:**
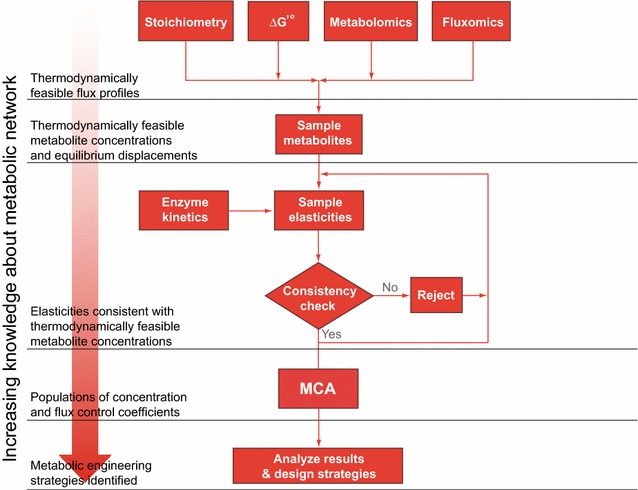
Flowchart of the ORACLE framework. The successive application of computational procedures integrates biological information from different sources, thereby refining kinetic models and providing guidance for metabolic engineering

The optimization and risk analysis of complex living entities (ORACLE) methodology used in this contribution is based on the MCA paradigm. ORACLE is composed of several successive computational procedures, each of them bringing a new level of information about metabolic networks (Fig. 8). Conceptually, ORACLE is organized as follows:

#### Level 1

We first determine the stoichiometry of the metabolic network using biochemical data or genome reconstructions [64–70]. For organisms with incompletely sequenced genomes, the part of the stoichiometry concerning missing pathways or compounds can be hypothesized. We proceed further by integrating the available information from metabolomics and fluxomics analyses [71–73], and using the estimates of the standard free energy of reactions [74–77] we perform the thermodynamics-based flux balance analysis (TFA) [78, 79]. Integration of thermodynamics at this stage allows us to eliminate thermodynamically unfeasible reactions, to establish reaction directionality, and to compute thermodynamically feasible flux profiles.

#### Level 2

Accurate and consistent determination of the space of metabolite concentrations plays a significant role in appraising all viable kinetic models sharing the same steady-state flux profile for two reasons: (1) the metabolite concentration levels intrinsically affect the Gibbs free energy difference; consequently, these have to be consistent with the reaction feasibility and directionality of computed flux profiles; (2) the metabolite concentration levels are one of the factors determining local stability of the kinetic models [32]. Uniform random sampling techniques are commonly used to investigate the space of metabolite concentrations [80]. However, the volume reduction of the thermodynamically feasible metabolite concentration space becomes so important in higher dimensional metabolite networks that acceptance–rejection methods are prohibitively inefficient in generating feasible random samples [81]. Furthermore, the thermodynamically feasible metabolite concentration space is not in the form of a parallelotope (nor it is in the form of a simplex or a hypersphere), and consequently the transformation techniques for the generation of random samples are not applicable [82, 83]. Therefore, we recur to Monte Carlo Markov Chain methods to generate random samples with distributions approaching the uniform distribution asymptotically. When experimental measurements or estimates from experimental data under similar physiological conditions are available for some metabolites in the network [84–86], we integrate these data as bounds on metabolite levels for the sampling.

We use the levels of allowable metabolite concentrations along with the standard Gibbs energy of reaction, $\Delta G^\circ ,$ to compute the *equilibrium displacements of thermodynamically feasible reactions*, $\varGamma$. Knowledge about equilibrium allow us, before even going to kinetics, to discern the reactions that are near thermodynamic equilibrium, i.e., the reactions that are not potential targets for metabolic engineering. The generated pairs of the metabolite concentration levels and the equilibrium displacements will subsequently be called ‘equilibrium displacement profiles’ as they are intrinsically inseparable.

#### Level 3

We integrate the kinetic properties of enzymes at this level. Available kinetic data from literature are incorporated. Whenever kinetic information about enzymes is incomplete or missing, we sample to recover the missing kinetic data. This is performed either through sampling of enzyme states [31], or through sampling of the degree of saturation of the enzyme active site [32].

At this level, we also perform consistency checks and pruning. In these tests, we evaluate the local stability of the resulting kinetic models and the consistency with the experimental information. As a result of the computational procedures performed at this level, we obtain the populations of *elasticities consistent with thermodynamically feasible metabolite concentrations.*

#### Level 4

Using the information gathered in previous levels, we compute *populations of control coefficients* and store them. Only the control coefficients stemming from stable systems are retained. If at a later time experimental evidence about the control coefficients becomes available, e.g., the response of a metabolic flux to an increase of activity of an enzyme becomes known, we prune the samples of the population with inconsistent control coefficients.

#### Level 5

We perform statistical analysis and data mining on the populations of control coefficients in order to quantify the importance of the enzymes in possible metabolic engineering strategies [87–89]. Hypotheses about possible couplings within the metabolic network are postulated at this level as well.

All the above-mentioned levels include visualization of the obtained knowledge about the metabolic network.

### Turnover control coefficients

The turnover of a metabolite, tMet, can be defined as the sum of fluxes that produce, or alternatively as the sum of fluxes that consume, the metabolite: ${\text{tMet}} = \mathop \sum \nolimits_{i = 1}^{n} v_{i}$. Here, *n* represents the number of producing (or consuming) fluxes $v_{i}$. The turnover control coefficients can then be calculated as

$$ C_{q}^{\text{tMet}} = \frac{q}{\text{tMet}}\frac{{{\text{dtMet}} }}{{{\text{d}}q}} = \mathop \sum \limits_{i = 1}^{n} \frac{{v_{i} }}{\text{tMet}}C_{q}^{{v_{i} }} , $$

where *q* denotes the set of system parameters, such as the enzyme activity and extracellular metabolite concentrations, and $C_{q}^{{v_{i} }}$ is the flux control coefficient of the flux $v_{i}$.

## Additional files

**Additional file 1.** Stoichiometry of used reactions and the corresponding mass balances.

**Additional file 2.** Thermodynamic displacement of reactions, Γ. Reactions can operate: (i) strictly far from thermodynamic equilibrium, i.e. 0 < Γ ≤ 0.1; (ii) with the middle displacements, i.e. 0.1 ≤ Γ ≤ 0.9; and (iii) strictly near equilibrium, i.e. 0.9 ≤ Γ < 1. Reactions whose displacements spanned more than one of these ranges were denoted with: (iv) I + II, for 0 < Γ ≤ 0.9; (v) II + III, for 0.1 ≤ Γ < 1; and (vi) I + II + III, for 0 < Γ < 1.

**Additional file 3.** List of reactions, their substrates and products, together with the used kinetic mechanisms (Additional file 10).

**Additional file 4.** Sheet “Flux Control Coefficients”: (i) Control coefficients of the xylose uptake (XTR) for a set of most important enzymes; (ii) Correlation of the distributions of the XTR control coefficients for HXK, ATPM and ZWF; (iii) The effects of manipulations of HXK, ATPM and NDR on metabolic fluxes. Sheet “Concentration Control Coeffs”: Control coefficients of the cytosolic ATP, cytosolic NADH and cytosolic NADPH during glucose-xylose co-utilization. Sheet “Turnover Control Coefficients”: (i) Turnover control coefficients of cytosolic ATP, mitochondrial ATP, cytosolic NADH, cytosolic NADPH, cytosolic protons and mitochondrial protons for HXK, ATPM and NDR; (ii) Flux control coefficients of producing and consuming reactions of cytosolic ATP, mitochondrial ATP, cytosolic NADH, cytosolic NADPH, cytosolic protons and mitochondrial protons for HXK, ATPM and NDR.

**Additional file 5.** Control coefficients of the flux through xylulose kinase (XK) during glucose-xylose co-utilisation for the original (purple) and refined (green) kinetic models. The bars represent the mean values of the control coefficients, and the error bars denote the 1st and the 3rd quartile of the control coefficients with respect to their mean value, i.e. 50% of the samples closest to the mean value are within the error bars.

**Additional file 6.** Predicted effects of changes in ATPM on the metabolic fluxes. The increased ATPM would result in reactions with: increased flux (red), decreased flux (green) or negligible changes in flux (grey). Higher intensity of red or green indicates larger changes in fluxes. The numerical values shown above reactions denote the mean control coefficients upon changes in ATPM, i.e. $C_{\text{ATPM}}^{*}$ where * denotes any metabolic flux in the network.

**Additional file 7.** Predicted effects of NDR manipulations on the metabolic fluxes. The increased activity of NDR would result in reactions with: increased flux (red), decreased flux (green) or negligible changes in flux (grey). Higher intensity of red or green indicates larger changes in fluxes. The numerical values shown above reactions denote the mean control coefficients upon activity changes in NDR, i.e. $C_{\text{NDR}}^{*}$ where * denotes any metabolic flux in the network.

**Additional file 8.** Flux Variability Analysis (FVA): (i) obtained ranges of the fluxes of reactions involved in the cytosolic ATP and NADPH metabolism for 4 different values of NDR flux; and (ii) scatter plots of the fluxes of reactions involved in the cytosolic ATP and NADPH metabolism for 4 different values of NDR flux.

**Additional file 9.** Estimated parameter values of the kinetic model used to smooth the measurement data together with the raw data (dots) and fitted model outputs (lines) for the cultivation of the VTT C-10880 and *HXK2* deletion strains. Correlation parameters for the measurement data and simulated values are also provided.

**Additional file 10.** Rate expressions for used kinetic mechanisms and the expressions for the corresponding metabolite elasticities.

## Abbreviations

ORACLE: optimization and risk analysis of complex living entities
TFA: thermodynamics-based flux balance analysis
*S. cerevisiae*: *Saccharomyces cerevisiae*
XTR: hexose transporters for xylose
XRI and XRII: xylose reductase
XLT: xylitol excretion transporter
XDH: xylitol reductase
XK: xylulokinase
GTR: hexose transporters for glucose
HXK: hexokinase
PGI: glucose-6-phosphate isomerase
PFK: phosphofructokinase
FBA: fructose-bisphosphate aldolase
TPI: triose phosphate isomerase
TDH: glyceraldehyde-3-phosphate dehydrogenase
PGK: phosphoglycerate kinase
GPM: phosphoglycerate mutase
ENO: enolase
PYK: pyruvate kinase
ZWF: glucose-6-phosphate-1-dehydrogenase
RKI: ribose-5-phosphate isomerase
RPE: ribulose-5-phosphate 3-epimerase
TKL1: transketolase
TKL2: transketolase
TAL: transaldolase
PDC: pyruvate decarboxylase
ALD: aldehyde dehydrogenase
ACS: acetyl-CoA synthase
CAT: carnitine o-acetyltransferase
ACARt: acetylcarnitine diffusion
YAT: carnitine o-acetyltransferase
CARt: carnitine diffusion
PYRt: pyruvate carrier
PDA: pyruvate dehydrogenase
PYC: pyruvate carboxylase
PCK: phosphoenolpyruvate carboxykinase
OAtrans: oxaloacetate carrier
MAE: malic enzyme
CIT: citrate synthase
ACO: aconitase
IDH: isocitrate dehydrogenase
KGD: a-ketoglutarate dehydrogenase
LSC: succinate-CoA ligase
SDH: succinate dehydrogenase
FUM: fumarase
MDH: malate dehydrogenase
NDH: external NADH dehydrogenase
NDI: NADH dehydrogenase
FDH: FADH2 dehydrogenase
NDR: NADPH reductase
QCR: ubiquinol cytochrome C reductase
COX: cytochrome C oxidase
ASN: ATP synthase
AAC: ADP/ATP carrier protein
ADK: adenylate kinase
ATPmt: ATP maintenance
ADH: cytosolic alcohol dehydrogenase
SCD: succinate dehydrogenase (ubiquinone-6), mitochondrial
ACET: acetate diffusion
COH: carbonic acid hydro-lyase
PPP: Pyrophosphate phosphohydrolase
MLPIT: malate transport, mitochondrial
ICL: Isocitrate glyoxylate-lyase
MLS: l-malate glyoxylate-lyase (CoA-acetylating)
MDHc: (S)-malate:NAD+ oxidoreductase
CITc: citrate oxaloacetate-lyase cytosolic
ACOc: citrate hydro-lyase cytosolic
LACm2r: d-lactate transport, mitochondrial
CITt2m: citrate transport, mitochondrial
LDH: (R)-lactate:ferricytochrome-c 2-oxidoreductase
O_2_m: O_2_ transport (diffusion)
CO_2_m: CO_2_ transport (diffusion), mitochondrial
PIm: phosphate transporter, mitochondrial
CO_2_t: CO_2_ transport via diffusion
GLYCt: glycerol transport in/out via diffusion reversible
PYRst: pyruvate transport via proton symport
SO_4_t: sulfate transport via proton symport
Pit: phosphate transport via proton symport
O_2_t: O_2_ transport via diffusion
NH_4_t: ammonia transport via diffusion
LACt2r: d-lactate transport via proton symport
SUCCt2r: succinate transporter in/out via proton symport
MALt2r: l-malate transport in via proton symport
GND1: 6-phospho-d-glucono-1,5-lactone lactonohydrolase
GND2: 6-phospho-d-gluconate:NADP+ 2-oxidoreductase (decarboxylating)
GPD1: glycerol-3-phosphate:NAD+ 2-oxidoreductase
GPD2: glycerol-3-phosphate phosphohydrolase
XL: xylose
XLT: xylitol
XYLL: xylulose
GLC: glucose
G6P: glucose-6-phosphate
F6P: fructose-6-phosphate
FBP: fructose 1,6-diphosphate
T3P: glyceraldehydes-3-phosphate
DHAP: glycerone phosphate
DPG: bisphosphoglycerate
3PG: 3-phosphoglycerate
2PG: 2-phosphoglycerate
PEP: phosphoenolpyruvate
PYR: pyruvate
6PGL: glucono-1,5-lactone 6-phosphate
RL5P: ribulose 5-phosphate
R5P: ribose 5-phosphate
X5P: xylose-5-phosphate
E4P: erythrose 4-phosphate
S7P: sedoheptulose 7-phosphate
AALD: acetaldehyde
ACET: acetate
ACCOA: acetyl-CoA
CAR: carnitine
ACAR: acetylcarnitine
OAA: oxaloacetate
CIT: citrate
ICIT: isocitrate
AKG: 2-oxoglutarate
SUCCOA: succinyl-CoA
SUCC: succinate
FUM: fumarate
MAL: malate
GL: glycerol
ETH: ethanol
PPi: pyrophosphate
Pi: phosphate
6PGC: 6-phospho-d-gluconate
GLYC3P: glycerol-3-phosphate
ACET: acetate
LAC: d-lactate
GLYX: glyoxylate

## Footnotes

1 Matlab files containing sets of computed flux and concentration control coefficients together with sampled metabolite concentrations, steady-state reference flux profile and stoichiometric matrix are available at https://doi.org/10.6084/m9.figshare.5028770.
