# Supplementary material for: A design–build–test cycle using modeling and experiments reveals interdependencies between upper glycolysis and xylose uptake in recombinant S. cerevisiae and improves predictive capabilities of large-scale kinetic models
Source: Biotechnol Biofuels. 2017 Jun 26;10:166. doi: 10.1186/s13068-017-0838-5 (PMC5485749; doi:10.1186/s13068-017-0838-5)
Supplement: Supplementary file 1 — Additional file 1. Stoichiometry of used reactions and the corresponding mass balances. [file 13068_2017_838_MOESM1_ESM.htm]

Reaction List and Mass Balances

# Reaction List

  
  
 1      **BIO**:      
  
  35.7018\*atp\_c  +   0.573604\*pyr\_c  +   0.123153\*nad\_c  +   1.85643\*g6p\_c  +   2.45575\*oaa\_c  +   0.889255\*f6p\_c  +   9.35735\*nadph\_c  +   5.19119\*nh4\_c  +   0.315171\*o2\_c  +   0.34124\*r5p\_c  +   0.47374\*3pg\_c  +   0.927128\*akg\_m  +   1.75986\*accoa\_c  +   0.481038\*pep\_c  +   0.240519\*e4p\_c  +   0.052144\*so4\_c    <-->    35.7018\*adp\_c    +    38.4641\*pi\_c    +    28.5469\*h\_c    +    2.3895\*co2\_c    +    0.123153\*nadh\_c    +    9.35735\*nadp\_c    +    0.064687\*t3p\_c    +    1.75986\*coa\_c    +    0.57662\*ppi\_c    +    0.0983\*mal\_c    
  
 2      **HXT**:      
  
  <-->    glc\_c    
  
 3      **HXK**:      
  
  atp\_c  +   glc\_c    <-->    g6p\_c    +    adp\_c    
  
 4      **PGI**:      
  
  g6p\_c    <-->    f6p\_c    
  
 5      **PFK**:      
  
  atp\_c  +   f6p\_c    <-->    adp\_c    +    fbp\_c    
  
 6      **FBA**:      
  
  fbp\_c    <-->    t3p\_c    +    dhap\_c    
  
 7      **TPI**:      
  
  dhap\_c    <-->    t3p\_c    
  
 8      **ZWF**:      
  
  g6p\_c  +   nadp\_c    <-->    nadph\_c    +    h\_c    +    6pgl\_c    
  
 9      **GND1**:      
  
  6pgl\_c    <-->    h\_c    +    6pgc\_c    
  
10      **GND2**:      
  
  nadp\_c  +   6pgc\_c    <-->    nadph\_c    +    co2\_c    +    rl5p\_c    
  
11      **RPE**:      
  
  rl5p\_c    <-->    x5p\_c    
  
12      **TKL2**:      
  
  e4p\_c  +   x5p\_c    <-->    f6p\_c    +    t3p\_c    
  
13      **TAL**:      
  
  t3p\_c  +   s7p\_c    <-->    f6p\_c    +    e4p\_c    
  
14      **RKI**:      
  
  rl5p\_c    <-->    r5p\_c    
  
15      **TKL1**:      
  
  r5p\_c  +   x5p\_c    <-->    t3p\_c    +    s7p\_c    
  
16      **TDH**:      
  
  nad\_c  +   pi\_c  +   t3p\_c    <-->    nadh\_c    +    dpg\_c    
  
17      **PGK**:      
  
  adp\_c  +   dpg\_c    <-->    atp\_c    +    3pg\_c    +    h\_c    
  
18      **GPM**:      
  
  3pg\_c    <-->    2pg\_c    
  
19      **ENO**:      
  
  2pg\_c    <-->    pep\_c    
  
20      **PEPCK**:      
  
  atp\_c  +   oaa\_c  +   h\_c    <-->    pep\_c    +    adp\_c    +    co2\_c    
  
21      **PYK**:      
  
  pep\_c  +   adp\_c    <-->    atp\_c    +    pyr\_c    
  
22      **PDC**:      
  
  pyr\_c  +   h\_c    <-->    co2\_c    +    aald\_c    
  
23      **ALD**:      
  
  nad\_c  +   aald\_c    <-->    2\*h\_c    +    nadh\_c    +    acet\_c    
  
24      **ATPM**:      
  
  atp\_c    <-->    adp\_c    +    pi\_c    +    h\_c    
  
25      **PYC**:      
  
  atp\_c  +   pyr\_c  +   hco3\_c    <-->    oaa\_c    +    adp\_c    +    pi\_c    +    h\_c    
  
26      **OAt**:      
  
  oaa\_c  +   h\_c    <-->    oaa\_m    +    h\_m    
  
27      **PYRm**:      
  
  pyr\_c  +   h\_c    <-->    pyr\_m    +    h\_m    
  
28      **PDA**:      
  
  pyr\_m  +   nad\_m  +   coa\_m    <-->    accoa\_m    +    co2\_m    +    nadh\_m    
  
29      **CIT**:      
  
  oaa\_m  +   accoa\_m    <-->    h\_m    +    coa\_m    +    cit\_m    
  
30      **ACO**:      
  
  cit\_m    <-->    icit\_m    
  
31      **IDH**:      
  
  nad\_m  +   icit\_m    <-->    akg\_m    +    co2\_m    +    nadh\_m    
  
32      **MDH**:      
  
  nad\_m  +   mal\_m    <-->    oaa\_m    +    h\_c    +    nadh\_m    
  
33      **MAE**:      
  
  nad\_m  +   mal\_m    <-->    pyr\_m    +    co2\_m    +    nadh\_m    
  
34      **NDH**:      
  
  h\_c  +   nadh\_c  +   q\_m    <-->    nad\_c    +    qh2\_m    
  
35      **NDI**:      
  
  h\_m  +   nadh\_m  +   q\_m    <-->    nad\_m    +    qh2\_m    
  
36      **QCR**:      
  
  1.5\*h\_m  +   qh2\_m  +   2\*feri\_m    <-->    3.5\*h\_c    +    q\_m    +    2\*fero\_m    
  
37      **COX**:      
  
  o2\_m  +   6\*h\_m  +   4\*fero\_m    <-->    2\*h\_c    +    4\*feri\_m    
  
38      **ASN**:      
  
  adp\_m  +   pi\_m  +   3\*h\_c    <-->    atp\_m    +    2\*h\_m    
  
39      **GPD1**:      
  
  h\_c  +   nadh\_c  +   dhap\_c    <-->    nad\_c    +    g3p\_c    
  
40      **GPD2**:      
  
  g3p\_c    <-->    pi\_c    +    h\_c    +    glyc\_c    
  
41      **ADH1**:      
  
  h\_c  +   nadh\_c  +   aald\_c    <-->    nad\_c    +    etoh\_c    
  
42      **ACE**:      
  
  h\_c  +   acet\_c    <-->    
  
43      **CAT**:      
  
  accoa\_c  +   car\_c    <-->    coa\_c    +    acar\_c    
  
44      **ACARt**:      
  
  acar\_c    <-->    acar\_m    
  
45      **YAT**:      
  
  coa\_m  +   acar\_m    <-->    accoa\_m    +    car\_m    
  
46      **CARt**:      
  
  car\_m    <-->    car\_c    
  
47      **ADK**:      
  
  atp\_c  +   amp\_c    <-->    2\*adp\_c    
  
48      **AAC**:      
  
  atp\_m  +   adp\_c  +   h\_c    <-->    atp\_c    +    adp\_m    +    h\_m    
  
49      **ACS**:      
  
  atp\_c  +   h\_c  +   coa\_c  +   acet\_c    <-->    accoa\_c    +    ppi\_c    +    amp\_c    
  
50      **LSC**:      
  
  adp\_m  +   pi\_m  +   succoa\_m    <-->    atp\_m    +    coa\_m    +    suc\_m    
  
51      **SCD**:      
  
  q\_m  +   suc\_m    <-->    qh2\_m    +    fum\_m    
  
52      **FUM**:      
  
  fum\_m    <-->    mal\_m    
  
53      **KGD**:      
  
  nad\_m  +   akg\_m  +   coa\_m    <-->    co2\_m    +    nadh\_m    +    succoa\_m    
  
54      **PIt**:      
  
  <-->    pi\_c    +    h\_c    
  
55      **COH**:      
  
  co2\_c    <-->    h\_c    +    hco3\_c    
  
56      **CO2t**:      
  
  co2\_c    <-->    
  
57      **O2t**:      
  
  <-->    o2\_c    
  
58      **GLYCt**:      
  
  glyc\_c    <-->    
  
59      **ETOHt**:      
  
  etoh\_c    <-->    
  
60      **O2m**:      
  
  o2\_c    <-->    o2\_m    
  
61      **CO2m**:      
  
  co2\_m    <-->    co2\_c    
  
62      **PIm**:      
  
  pi\_c  +   h\_c    <-->    pi\_m    +    h\_m    
  
63      **PPP**:      
  
  ppi\_c    <-->    2\*pi\_c    +    2\*h\_c    
  
64      **NDR**:      
  
  nadp\_c  +   2\*feri\_m    <-->    nadph\_c    +    2\*fero\_m    
  
65      **NH4t**:      
  
  <-->    nh4\_c    
  
66      **SO4t**:      
  
  <-->    so4\_c    +    h\_c    
  
67      **MLPIT**:      
  
  pi\_c  +   mal\_m    <-->    pi\_m    +    mal\_c    
  
68      **MDHc**:      
  
  nad\_c  +   mal\_c    <-->    oaa\_c    +    h\_c    +    nadh\_c    
  
69      **CITc**:      
  
  h\_c  +   coa\_c  +   cit\_c    <-->    oaa\_c    +    accoa\_c    
  
70      **ACOc**:      
  
  cit\_c    <-->    icit\_c    
  
71      **ICL**:      
  
  icit\_c    <-->    suc\_c    +    glyx\_c    
  
72      **MLS**:      
  
  accoa\_c  +   glyx\_c    <-->    h\_c    +    coa\_c    +    mal\_c    
  
73      **XTR**:      
  
  <-->    xyl\_c    
  
74      **XDH**:      
  
  nad\_c  +   xlt\_c    <-->    h\_c    +    nadh\_c    +    xyll\_c    
  
75      **XRI**:      
  
  h\_c  +   nadh\_c  +   xyl\_c    <-->    nad\_c    +    xlt\_c    
  
76      **XRII**:      
  
  nadph\_c  +   h\_c  +   xyl\_c    <-->    nadp\_c    +    xlt\_c    
  
77      **XK**:      
  
  atp\_c  +   xyll\_c    <-->    adp\_c    +    x5p\_c    
  
78      **XLT**:      
  
  xlt\_c    <-->    
  
79      **D\_LACt2r**:      
  
  h\_c  +   lac\_c    <-->    
  
80      **D\_LACm2r**:      
  
  h\_m  +   lac\_m    <-->    h\_c    +    lac\_c    
  
81      **D\_LAC**:      
  
  pyr\_m  +   2\*h\_m  +   2\*fero\_m    <-->    2\*feri\_m    +    lac\_m    
  
82      **SUCCt2r**:      
  
  h\_c  +   suc\_c    <-->    
  
83      **MALt2r**:      
  
  h\_c  +   mal\_c    <-->    
  
84      **CITt2m**:      
  
  mal\_c  +   cit\_m    <-->    mal\_m    +    cit\_c    
  
  
  

# Mass Balances

  
  
 1      d/dt( atp\_c )   =    PGK    +    PYK    +    AAC  -   35.7018\*  BIO  -   HXK  -   PFK  -   PEPCK  -   ATPM  -   PYC  -   ADK  -   ACS  -   XK    
  
 2      d/dt( atp\_m )   =    ASN    +    LSC  -   AAC    
  
 3      d/dt( pyr\_c )   =    PYK  -   0.573604\*  BIO  -   PDC  -   PYC  -   PYRm    
  
 4      d/dt( pyr\_m )   =    PYRm    +    MAE  -   PDA  -   D\_LAC    
  
 5      d/dt( nad\_c )   =    NDH    +    GPD1    +    ADH1    +    XRI  -   0.123153\*  BIO  -   TDH  -   ALD  -   MDHc  -   XDH    
  
 6      d/dt( nad\_m )   =    NDI  -   PDA  -   IDH  -   MDH  -   MAE  -   KGD    
  
 7      d/dt( g6p\_c )   =    HXK  -   1.85643\*  BIO  -   PGI  -   ZWF    
  
 8      d/dt( oaa\_c )   =    PYC    +    MDHc    +    CITc  -   2.45575\*  BIO  -   PEPCK  -   OAt    
  
 9      d/dt( oaa\_m )   =    OAt    +    MDH  -   CIT    
  
10      d/dt( f6p\_c )   =    PGI    +    TKL2    +    TAL  -   0.889255\*  BIO  -   PFK    
  
11      d/dt( nadph\_c )   =    ZWF    +    GND2    +    NDR  -   9.35735\*  BIO  -   XRII    
  
12      d/dt( nh4\_c )   =    NH4t  -   5.19119\*  BIO    
  
13      d/dt( o2\_c )   =    O2t  -   0.315171\*  BIO  -   O2m    
  
14      d/dt( o2\_m )   =    O2m  -   COX    
  
15      d/dt( r5p\_c )   =    RKI  -   0.34124\*  BIO  -   TKL1    
  
16      d/dt( 3pg\_c )   =    PGK  -   0.47374\*  BIO  -   GPM    
  
17      d/dt( akg\_m )   =    IDH  -   0.927128\*  BIO  -   KGD    
  
18      d/dt( accoa\_c )   =    ACS    +    CITc  -   1.75986\*  BIO  -   CAT  -   MLS    
  
19      d/dt( accoa\_m )   =    PDA    +    YAT  -   CIT    
  
20      d/dt( pep\_c )   =    ENO    +    PEPCK  -   0.481038\*  BIO  -   PYK    
  
21      d/dt( e4p\_c )   =    TAL  -   0.240519\*  BIO  -   TKL2    
  
22      d/dt( so4\_c )   =    SO4t  -   0.052144\*  BIO    
  
23      d/dt( adp\_c )   =    35.7018\*  BIO    +    HXK    +    PFK    +    PEPCK    +    ATPM    +    PYC    +    2\*  ADK    +    XK  -   PGK  -   PYK  -   AAC    
  
24      d/dt( adp\_m )   =    AAC  -   ASN  -   LSC    
  
25      d/dt( pi\_c )   =    38.4641\*  BIO    +    ATPM    +    PYC    +    GPD2    +    PIt    +    2\*  PPP  -   TDH  -   PIm  -   MLPIT    
  
26      d/dt( pi\_m )   =    PIm    +    MLPIT  -   ASN  -   LSC    
  
27      d/dt( h\_c )   =    28.5469\*  BIO    +    ZWF    +    GND1    +    PGK    +    2\*  ALD    +    ATPM    +    PYC    +    MDH    +    3.5\*  QCR    +    2\*  COX    +    GPD2    +    PIt    +    COH    +    2\*  PPP    +    SO4t    +    MDHc    +    MLS    +    XDH    +    D\_LACm2r  -   PEPCK  -   PDC  -   OAt  -   PYRm  -   NDH  -   3\*  ASN  -   GPD1  -   ADH1  -   ACE  -   AAC  -   ACS  -   PIm  -   CITc  -   XRI  -   XRII  -   D\_LACt2r  -   SUCCt2r  -   MALt2r    
  
28      d/dt( h\_m )   =    OAt    +    PYRm    +    CIT    +    2\*  ASN    +    AAC    +    PIm  -   NDI  -   1.5\*  QCR  -   6\*  COX  -   D\_LACm2r  -   2\*  D\_LAC    
  
29      d/dt( co2\_c )   =    2.3895\*  BIO    +    GND2    +    PEPCK    +    PDC    +    CO2m  -   COH  -   CO2t    
  
30      d/dt( co2\_m )   =    PDA    +    IDH    +    MAE    +    KGD  -   CO2m    
  
31      d/dt( nadh\_c )   =    0.123153\*  BIO    +    TDH    +    ALD    +    MDHc    +    XDH  -   NDH  -   GPD1  -   ADH1  -   XRI    
  
32      d/dt( nadh\_m )   =    PDA    +    IDH    +    MDH    +    MAE    +    KGD  -   NDI    
  
33      d/dt( nadp\_c )   =    9.35735\*  BIO    +    XRII  -   ZWF  -   GND2  -   NDR    
  
34      d/dt( t3p\_c )   =    0.064687\*  BIO    +    FBA    +    TPI    +    TKL2    +    TKL1  -   TAL  -   TDH    
  
35      d/dt( coa\_c )   =    1.75986\*  BIO    +    CAT    +    MLS  -   ACS  -   CITc    
  
36      d/dt( coa\_m )   =    CIT    +    LSC  -   PDA  -   YAT  -   KGD    
  
37      d/dt( ppi\_c )   =    0.57662\*  BIO    +    ACS  -   PPP    
  
38      d/dt( mal\_c )   =    0.0983\*  BIO    +    MLPIT    +    MLS  -   MDHc  -   MALt2r  -   CITt2m    
  
39      d/dt( mal\_m )   =    FUM    +    CITt2m  -   MDH  -   MAE  -   MLPIT    
  
40      d/dt( glc\_c )   =    HXT  -   HXK    
  
41      d/dt( fbp\_c )   =    PFK  -   FBA    
  
42      d/dt( dhap\_c )   =    FBA  -   TPI  -   GPD1    
  
43      d/dt( 6pgl\_c )   =    ZWF  -   GND1    
  
44      d/dt( 6pgc\_c )   =    GND1  -   GND2    
  
45      d/dt( rl5p\_c )   =    GND2  -   RPE  -   RKI    
  
46      d/dt( x5p\_c )   =    RPE    +    XK  -   TKL2  -   TKL1    
  
47      d/dt( s7p\_c )   =    TKL1  -   TAL    
  
48      d/dt( dpg\_c )   =    TDH  -   PGK    
  
49      d/dt( 2pg\_c )   =    GPM  -   ENO    
  
50      d/dt( aald\_c )   =    PDC  -   ALD  -   ADH1    
  
51      d/dt( acet\_c )   =    ALD  -   ACE  -   ACS    
  
52      d/dt( hco3\_c )   =    COH  -   PYC    
  
53      d/dt( cit\_c )   =    CITt2m  -   CITc  -   ACOc    
  
54      d/dt( cit\_m )   =    CIT  -   ACO  -   CITt2m    
  
55      d/dt( icit\_c )   =    ACOc  -   ICL    
  
56      d/dt( icit\_m )   =    ACO  -   IDH    
  
57      d/dt( q\_m )   =    QCR  -   NDH  -   NDI  -   SCD    
  
58      d/dt( qh2\_m )   =    NDH    +    NDI    +    SCD  -   QCR    
  
59      d/dt( feri\_m )   =    4\*  COX    +    2\*  D\_LAC  -   2\*  QCR  -   2\*  NDR    
  
60      d/dt( fero\_m )   =    2\*  QCR    +    2\*  NDR  -   4\*  COX  -   2\*  D\_LAC    
  
61      d/dt( g3p\_c )   =    GPD1  -   GPD2    
  
62      d/dt( glyc\_c )   =    GPD2  -   GLYCt    
  
63      d/dt( etoh\_c )   =    ADH1  -   ETOHt    
  
64      d/dt( car\_c )   =    CARt  -   CAT    
  
65      d/dt( car\_m )   =    YAT  -   CARt    
  
66      d/dt( acar\_c )   =    CAT  -   ACARt    
  
67      d/dt( acar\_m )   =    ACARt  -   YAT    
  
68      d/dt( amp\_c )   =    ACS  -   ADK    
  
69      d/dt( suc\_c )   =    ICL  -   SUCCt2r    
  
70      d/dt( suc\_m )   =    LSC  -   SCD    
  
71      d/dt( succoa\_m )   =    KGD  -   LSC    
  
72      d/dt( fum\_m )   =    SCD  -   FUM    
  
73      d/dt( glyx\_c )   =    ICL  -   MLS    
  
74      d/dt( xyl\_c )   =    XTR  -   XRI  -   XRII    
  
75      d/dt( xlt\_c )   =    XRI    +    XRII  -   XDH  -   XLT    
  
76      d/dt( xyll\_c )   =    XDH  -   XK    
  
77      d/dt( lac\_c )   =    D\_LACm2r  -   D\_LACt2r    
  
78      d/dt( lac\_m )   =    D\_LAC  -   D\_LACm2r    
