# Supplementary material for: A design–build–test cycle using modeling and experiments reveals interdependencies between upper glycolysis and xylose uptake in recombinant S. cerevisiae and improves predictive capabilities of large-scale kinetic models
Source: Biotechnol Biofuels. 2017 Jun 26;10:166. doi: 10.1186/s13068-017-0838-5 (PMC5485749; doi:10.1186/s13068-017-0838-5)
Supplement: Supplementary file 3 — Additional file 3. List of reactions, their substrates and products, together with the used kinetic mechanisms (Additional file 10). [file 13068_2017_838_MOESM3_ESM.htm]

Kinetic Data of Reactions

# Kinetic Data of Reactions

  
  
1      **BIO**:  
  
  
          35.7018\*atp\_c   +    0.573604\*pyr\_c   +    0.123153\*nad\_c   +    1.85643\*g6p\_c   +    2.45575\*oaa\_c   +    0.889255\*f6p\_c   +    9.35735\*nadph\_c   +    5.19119\*nh4\_c   +    0.315171\*o2\_c   +    0.34124\*r5p\_c   +    0.47374\*3pg\_c   +    0.927128\*akg\_m   +    1.75986\*accoa\_c   +    0.481038\*pep\_c   +    0.240519\*e4p\_c   +    0.052144\*so4\_c    <-->    35.7018\*adp\_c    +     38.4641\*pi\_c    +     28.5469\*h\_c    +     2.3895\*co2\_c    +     0.123153\*nadh\_c    +     9.35735\*nadp\_c    +     0.064687\*t3p\_c    +     1.75986\*coa\_c    +     0.57662\*ppi\_c    +     0.0983\*mal\_c     
  
  
  Substrates:   
  
      
   atp\_c,   pyr\_c,   nad\_c,   g6p\_c,   oaa\_c,   f6p\_c,   nadph\_c,   nh4\_c,   o2\_c,   r5p\_c,   3pg\_c,   akg\_m,   accoa\_c,   pep\_c,   e4p\_c,   so4\_c
  
  
  Products:   
  
      
   adp\_c,   pi\_c,   h\_c,   co2\_c,   nadh\_c,   nadp\_c,   t3p\_c,   coa\_c,   ppi\_c,   mal\_c  
  
  Kinetic mechanism:   BS16   
  
  


---

  
  
2      **HXT**:  
  
  
        <-->    glc\_c     
  
  
  Substrates:   
  
      
   N/A
  
  
  Products:   
  
      
   glc\_c  
  
  Kinetic mechanism:   A1   
  
  


---

  
  
3      **HXK**:  
  
  
          atp\_c   +    glc\_c    <-->    g6p\_c    +     adp\_c     
  
  
  Substrates:   
  
      
   atp\_c,   glc\_c
  
  
  Products:   
  
      
   g6p\_c,   adp\_c  
  
  Kinetic mechanism:   A2   
  
  


---

  
  
4      **PGI**:  
  
  
          g6p\_c    <-->    f6p\_c     
  
  
  Substrates:   
  
      
   g6p\_c
  
  
  Products:   
  
      
   f6p\_c  
  
  Kinetic mechanism:   A1   
  
  


---

  
  
5      **PFK**:  
  
  
          atp\_c   +    f6p\_c    <-->    adp\_c    +     fbp\_c     
  
  
  Substrates:   
  
      
   atp\_c,   f6p\_c
  
  
  Products:   
  
      
   adp\_c,   fbp\_c  
  
  Kinetic mechanism:   A99   
  
  


---

  
  
6      **FBA**:  
  
  
          fbp\_c    <-->    t3p\_c    +     dhap\_c     
  
  
  Substrates:   
  
      
   fbp\_c
  
  
  Products:   
  
      
   t3p\_c,   dhap\_c  
  
  Kinetic mechanism:   RHUB   
  
  


---

  
  
7      **TPI**:  
  
  
          dhap\_c    <-->    t3p\_c     
  
  
  Substrates:   
  
      
   dhap\_c
  
  
  Products:   
  
      
   t3p\_c  
  
  Kinetic mechanism:   A1   
  
  


---

  
  
8      **ZWF**:  
  
  
          g6p\_c   +    nadp\_c    <-->    nadph\_c    +     h\_c    +     6pgl\_c     
  
  
  Substrates:   
  
      
   g6p\_c,   nadp\_c
  
  
  Products:   
  
      
   nadph\_c,   h\_c,   6pgl\_c  
  
  Kinetic mechanism:   A2CSP   
  
  


---

  
  
9      **GND1**:  
  
  
          6pgl\_c    <-->    h\_c    +     6pgc\_c     
  
  
  Substrates:   
  
      
   6pgl\_c
  
  
  Products:   
  
      
   h\_c,   6pgc\_c  
  
  Kinetic mechanism:   A1CSP   
  
  


---

  
  
10      **GND2**:  
  
  
          nadp\_c   +    6pgc\_c    <-->    nadph\_c    +     co2\_c    +     rl5p\_c     
  
  
  Substrates:   
  
      
   nadp\_c,   6pgc\_c
  
  
  Products:   
  
      
   nadph\_c,   co2\_c,   rl5p\_c  
  
  Kinetic mechanism:   A2CSP   
  
  


---

  
  
11      **RPE**:  
  
  
          rl5p\_c    <-->    x5p\_c     
  
  
  Substrates:   
  
      
   rl5p\_c
  
  
  Products:   
  
      
   x5p\_c  
  
  Kinetic mechanism:   A1   
  
  


---

  
  
12      **TKL2**:  
  
  
          e4p\_c   +    x5p\_c    <-->    f6p\_c    +     t3p\_c     
  
  
  Substrates:   
  
      
   e4p\_c,   x5p\_c
  
  
  Products:   
  
      
   f6p\_c,   t3p\_c  
  
  Kinetic mechanism:   A2   
  
  


---

  
  
13      **TAL**:  
  
  
          t3p\_c   +    s7p\_c    <-->    f6p\_c    +     e4p\_c     
  
  
  Substrates:   
  
      
   t3p\_c,   s7p\_c
  
  
  Products:   
  
      
   f6p\_c,   e4p\_c  
  
  Kinetic mechanism:   A2   
  
  


---

  
  
14      **RKI**:  
  
  
          rl5p\_c    <-->    r5p\_c     
  
  
  Substrates:   
  
      
   rl5p\_c
  
  
  Products:   
  
      
   r5p\_c  
  
  Kinetic mechanism:   A1   
  
  


---

  
  
15      **TKL1**:  
  
  
          r5p\_c   +    x5p\_c    <-->    t3p\_c    +     s7p\_c     
  
  
  Substrates:   
  
      
   r5p\_c,   x5p\_c
  
  
  Products:   
  
      
   t3p\_c,   s7p\_c  
  
  Kinetic mechanism:   A2   
  
  


---

  
  
16      **TDH**:  
  
  
          nad\_c   +    pi\_c   +    t3p\_c    <-->    nadh\_c    +     dpg\_c     
  
  
  Substrates:   
  
      
   nad\_c,   pi\_c,   t3p\_c
  
  
  Products:   
  
      
   nadh\_c,   dpg\_c  
  
  Kinetic mechanism:   A2CSP   
  
  


---

  
  
17      **PGK**:  
  
  
          adp\_c   +    dpg\_c    <-->    atp\_c    +     3pg\_c    +     h\_c     
  
  
  Substrates:   
  
      
   adp\_c,   dpg\_c
  
  
  Products:   
  
      
   atp\_c,   3pg\_c,   h\_c  
  
  Kinetic mechanism:   A2CSP   
  
  


---

  
  
18      **GPM**:  
  
  
          3pg\_c    <-->    2pg\_c     
  
  
  Substrates:   
  
      
   3pg\_c
  
  
  Products:   
  
      
   2pg\_c  
  
  Kinetic mechanism:   A1   
  
  


---

  
  
19      **ENO**:  
  
  
          2pg\_c    <-->    pep\_c     
  
  
  Substrates:   
  
      
   2pg\_c
  
  
  Products:   
  
      
   pep\_c  
  
  Kinetic mechanism:   A1   
  
  


---

  
  
20      **PEPCK**:  
  
  
          atp\_c   +    oaa\_c   +    h\_c    <-->    pep\_c    +     adp\_c    +     co2\_c     
  
  
  Substrates:   
  
      
   atp\_c,   oaa\_c,   h\_c
  
  
  Products:   
  
      
   pep\_c,   adp\_c,   co2\_c  
  
  Kinetic mechanism:   A2CSP   
  
  


---

  
  
21      **PYK**:  
  
  
          pep\_c   +    adp\_c    <-->    atp\_c    +     pyr\_c     
  
  
  Substrates:   
  
      
   pep\_c,   adp\_c
  
  
  Products:   
  
      
   atp\_c,   pyr\_c  
  
  Kinetic mechanism:   A2   
  
  


---

  
  
22      **PDC**:  
  
  
          pyr\_c   +    h\_c    <-->    co2\_c    +     aald\_c     
  
  
  Substrates:   
  
      
   pyr\_c,   h\_c
  
  
  Products:   
  
      
   co2\_c,   aald\_c  
  
  Kinetic mechanism:   RHUB2CS   
  
  


---

  
  
23      **ALD**:  
  
  
          nad\_c   +    aald\_c    <-->    2\*h\_c    +     nadh\_c    +     acet\_c     
  
  
  Substrates:   
  
      
   nad\_c,   aald\_c
  
  
  Products:   
  
      
   h\_c,   nadh\_c,   acet\_c  
  
  Kinetic mechanism:   A2CPa   
  
  


---

  
  
24      **ATPM**:  
  
  
          atp\_c    <-->    adp\_c    +     pi\_c    +     h\_c     
  
  
  Substrates:   
  
      
   atp\_c
  
  
  Products:   
  
      
   adp\_c,   pi\_c,   h\_c  
  
  Kinetic mechanism:   A1CP2i   
  
  


---

  
  
25      **PYC**:  
  
  
          atp\_c   +    pyr\_c   +    hco3\_c    <-->    oaa\_c    +     adp\_c    +     pi\_c    +     h\_c     
  
  
  Substrates:   
  
      
   atp\_c,   pyr\_c,   hco3\_c
  
  
  Products:   
  
      
   oaa\_c,   adp\_c,   pi\_c,   h\_c  
  
  Kinetic mechanism:   A2CS2P2   
  
  


---

  
  
26      **OAt**:  
  
  
          oaa\_c   +    h\_c    <-->    oaa\_m    +     h\_m     
  
  
  Substrates:   
  
      
   oaa\_c,   h\_c
  
  
  Products:   
  
      
   oaa\_m,   h\_m  
  
  Kinetic mechanism:   A1CSP   
  
  


---

  
  
27      **PYRm**:  
  
  
          pyr\_c   +    h\_c    <-->    pyr\_m    +     h\_m     
  
  
  Substrates:   
  
      
   pyr\_c,   h\_c
  
  
  Products:   
  
      
   pyr\_m,   h\_m  
  
  Kinetic mechanism:   A1CSP   
  
  


---

  
  
28      **PDA**:  
  
  
          pyr\_m   +    nad\_m   +    coa\_m    <-->    accoa\_m    +     co2\_m    +     nadh\_m     
  
  
  Substrates:   
  
      
   pyr\_m,   nad\_m,   coa\_m
  
  
  Products:   
  
      
   accoa\_m,   co2\_m,   nadh\_m  
  
  Kinetic mechanism:   TBCSP   
  
  


---

  
  
29      **CIT**:  
  
  
          oaa\_m   +    accoa\_m    <-->    h\_m    +     coa\_m    +     cit\_m     
  
  
  Substrates:   
  
      
   oaa\_m,   accoa\_m
  
  
  Products:   
  
      
   h\_m,   coa\_m,   cit\_m  
  
  Kinetic mechanism:   A2CSP   
  
  


---

  
  
30      **ACO**:  
  
  
          cit\_m    <-->    icit\_m     
  
  
  Substrates:   
  
      
   cit\_m
  
  
  Products:   
  
      
   icit\_m  
  
  Kinetic mechanism:   A1   
  
  


---

  
  
31      **IDH**:  
  
  
          nad\_m   +    icit\_m    <-->    akg\_m    +     co2\_m    +     nadh\_m     
  
  
  Substrates:   
  
      
   nad\_m,   icit\_m
  
  
  Products:   
  
      
   akg\_m,   co2\_m,   nadh\_m  
  
  Kinetic mechanism:   A2CSP   
  
  


---

  
  
32      **MDH**:  
  
  
          nad\_m   +    mal\_m    <-->    oaa\_m    +     h\_c    +     nadh\_m     
  
  
  Substrates:   
  
      
   nad\_m,   mal\_m
  
  
  Products:   
  
      
   oaa\_m,   h\_c,   nadh\_m  
  
  Kinetic mechanism:   A2CSP   
  
  


---

  
  
33      **MAE**:  
  
  
          nad\_m   +    mal\_m    <-->    pyr\_m    +     co2\_m    +     nadh\_m     
  
  
  Substrates:   
  
      
   nad\_m,   mal\_m
  
  
  Products:   
  
      
   pyr\_m,   co2\_m,   nadh\_m  
  
  Kinetic mechanism:   A2CSP   
  
  


---

  
  
34      **NDH**:  
  
  
          h\_c   +    nadh\_c   +    q\_m    <-->    nad\_c    +     qh2\_m     
  
  
  Substrates:   
  
      
   h\_c,   nadh\_c,   q\_m
  
  
  Products:   
  
      
   nad\_c,   qh2\_m  
  
  Kinetic mechanism:   A2CSP   
  
  


---

  
  
35      **NDI**:  
  
  
          h\_m   +    nadh\_m   +    q\_m    <-->    nad\_m    +     qh2\_m     
  
  
  Substrates:   
  
      
   h\_m,   nadh\_m,   q\_m
  
  
  Products:   
  
      
   nad\_m,   qh2\_m  
  
  Kinetic mechanism:   A2CSP   
  
  


---

  
  
36      **QCR**:  
  
  
          1.5\*h\_m   +    qh2\_m   +    2\*feri\_m    <-->    3.5\*h\_c    +     q\_m    +     2\*fero\_m     
  
  
  Substrates:   
  
      
   h\_m,   qh2\_m,   feri\_m
  
  
  Products:   
  
      
   h\_c,   q\_m,   fero\_m  
  
  Kinetic mechanism:   A2CSPQCR   
  
  


---

  
  
37      **COX**:  
  
  
          o2\_m   +    6\*h\_m   +    4\*fero\_m    <-->    2\*h\_c    +     4\*feri\_m     
  
  
  Substrates:   
  
      
   o2\_m,   h\_m,   fero\_m
  
  
  Products:   
  
      
   h\_c,   feri\_m  
  
  Kinetic mechanism:   RHBUCOX   
  
  


---

  
  
38      **ASN**:  
  
  
          adp\_m   +    pi\_m   +    3\*h\_c    <-->    atp\_m    +     2\*h\_m     
  
  
  Substrates:   
  
      
   adp\_m,   pi\_m,   h\_c
  
  
  Products:   
  
      
   atp\_m,   h\_m  
  
  Kinetic mechanism:   RHBU3ASN   
  
  


---

  
  
39      **GPD1**:  
  
  
          h\_c   +    nadh\_c   +    dhap\_c    <-->    nad\_c    +     g3p\_c     
  
  
  Substrates:   
  
      
   h\_c,   nadh\_c,   dhap\_c
  
  
  Products:   
  
      
   nad\_c,   g3p\_c  
  
  Kinetic mechanism:   A2CSP   
  
  


---

  
  
40      **GPD2**:  
  
  
          g3p\_c    <-->    pi\_c    +     h\_c    +     glyc\_c     
  
  
  Substrates:   
  
      
   g3p\_c
  
  
  Products:   
  
      
   pi\_c,   h\_c,   glyc\_c  
  
  Kinetic mechanism:   RHUBCSP   
  
  


---

  
  
41      **ADH1**:  
  
  
          h\_c   +    nadh\_c   +    aald\_c    <-->    nad\_c    +     etoh\_c     
  
  
  Substrates:   
  
      
   h\_c,   nadh\_c,   aald\_c
  
  
  Products:   
  
      
   nad\_c,   etoh\_c  
  
  Kinetic mechanism:   A6CS   
  
  


---

  
  
42      **ACE**:  
  
  
          h\_c   +    acet\_c    <-->   
  
  
  Substrates:   
  
      
   h\_c,   acet\_c
  
  
  Products:   
  
      
   N/A  
  
  Kinetic mechanism:   A1CSP   
  
  


---

  
  
43      **CAT**:  
  
  
          accoa\_c   +    car\_c    <-->    coa\_c    +     acar\_c     
  
  
  Substrates:   
  
      
   accoa\_c,   car\_c
  
  
  Products:   
  
      
   coa\_c,   acar\_c  
  
  Kinetic mechanism:   A2   
  
  


---

  
  
44      **ACARt**:  
  
  
          acar\_c    <-->    acar\_m     
  
  
  Substrates:   
  
      
   acar\_c
  
  
  Products:   
  
      
   acar\_m  
  
  Kinetic mechanism:   A1   
  
  


---

  
  
45      **YAT**:  
  
  
          coa\_m   +    acar\_m    <-->    accoa\_m    +     car\_m     
  
  
  Substrates:   
  
      
   coa\_m,   acar\_m
  
  
  Products:   
  
      
   accoa\_m,   car\_m  
  
  Kinetic mechanism:   A2   
  
  


---

  
  
46      **CARt**:  
  
  
          car\_m    <-->    car\_c     
  
  
  Substrates:   
  
      
   car\_m
  
  
  Products:   
  
      
   car\_c  
  
  Kinetic mechanism:   A1   
  
  


---

  
  
47      **ADK**:  
  
  
          atp\_c   +    amp\_c    <-->    2\*adp\_c     
  
  
  Substrates:   
  
      
   atp\_c,   amp\_c
  
  
  Products:   
  
      
   adp\_c  
  
  Kinetic mechanism:   B4   
  
  


---

  
  
48      **AAC**:  
  
  
          atp\_m   +    adp\_c   +    h\_c    <-->    atp\_c    +     adp\_m    +     h\_m     
  
  
  Substrates:   
  
      
   atp\_m,   adp\_c,   h\_c
  
  
  Products:   
  
      
   atp\_c,   adp\_m,   h\_m  
  
  Kinetic mechanism:   A2CSP   
  
  


---

  
  
49      **ACS**:  
  
  
          atp\_c   +    h\_c   +    coa\_c   +    acet\_c    <-->    accoa\_c    +     ppi\_c    +     amp\_c     
  
  
  Substrates:   
  
      
   atp\_c,   h\_c,   coa\_c,   acet\_c
  
  
  Products:   
  
      
   accoa\_c,   ppi\_c,   amp\_c  
  
  Kinetic mechanism:   TBCSP   
  
  


---

  
  
50      **LSC**:  
  
  
          adp\_m   +    pi\_m   +    succoa\_m    <-->    atp\_m    +     coa\_m    +     suc\_m     
  
  
  Substrates:   
  
      
   adp\_m,   pi\_m,   succoa\_m
  
  
  Products:   
  
      
   atp\_m,   coa\_m,   suc\_m  
  
  Kinetic mechanism:   BTCSP   
  
  


---

  
  
51      **SCD**:  
  
  
          q\_m   +    suc\_m    <-->    qh2\_m    +     fum\_m     
  
  
  Substrates:   
  
      
   q\_m,   suc\_m
  
  
  Products:   
  
      
   qh2\_m,   fum\_m  
  
  Kinetic mechanism:   A2   
  
  


---

  
  
52      **FUM**:  
  
  
          fum\_m    <-->    mal\_m     
  
  
  Substrates:   
  
      
   fum\_m
  
  
  Products:   
  
      
   mal\_m  
  
  Kinetic mechanism:   A1   
  
  


---

  
  
53      **KGD**:  
  
  
          nad\_m   +    akg\_m   +    coa\_m    <-->    co2\_m    +     nadh\_m    +     succoa\_m     
  
  
  Substrates:   
  
      
   nad\_m,   akg\_m,   coa\_m
  
  
  Products:   
  
      
   co2\_m,   nadh\_m,   succoa\_m  
  
  Kinetic mechanism:   TBCSP   
  
  


---

  
  
54      **PIt**:  
  
  
        <-->    pi\_c    +     h\_c     
  
  
  Substrates:   
  
      
   N/A
  
  
  Products:   
  
      
   pi\_c,   h\_c  
  
  Kinetic mechanism:   A1CSP   
  
  


---

  
  
55      **COH**:  
  
  
          co2\_c    <-->    h\_c    +     hco3\_c     
  
  
  Substrates:   
  
      
   co2\_c
  
  
  Products:   
  
      
   h\_c,   hco3\_c  
  
  Kinetic mechanism:   CHEM   
  
  


---

  
  
56      **CO2t**:  
  
  
          co2\_c    <-->   
  
  
  Substrates:   
  
      
   co2\_c
  
  
  Products:   
  
      
   N/A  
  
  Kinetic mechanism:   A1   
  
  


---

  
  
57      **O2t**:  
  
  
        <-->    o2\_c     
  
  
  Substrates:   
  
      
   N/A
  
  
  Products:   
  
      
   o2\_c  
  
  Kinetic mechanism:   A1   
  
  


---

  
  
58      **GLYCt**:  
  
  
          glyc\_c    <-->   
  
  
  Substrates:   
  
      
   glyc\_c
  
  
  Products:   
  
      
   N/A  
  
  Kinetic mechanism:   A1   
  
  


---

  
  
59      **ETOHt**:  
  
  
          etoh\_c    <-->   
  
  
  Substrates:   
  
      
   etoh\_c
  
  
  Products:   
  
      
   N/A  
  
  Kinetic mechanism:   A1   
  
  


---

  
  
60      **O2m**:  
  
  
          o2\_c    <-->    o2\_m     
  
  
  Substrates:   
  
      
   o2\_c
  
  
  Products:   
  
      
   o2\_m  
  
  Kinetic mechanism:   A1   
  
  


---

  
  
61      **CO2m**:  
  
  
          co2\_m    <-->    co2\_c     
  
  
  Substrates:   
  
      
   co2\_m
  
  
  Products:   
  
      
   co2\_c  
  
  Kinetic mechanism:   A1   
  
  


---

  
  
62      **PIm**:  
  
  
          pi\_c   +    h\_c    <-->    pi\_m    +     h\_m     
  
  
  Substrates:   
  
      
   pi\_c,   h\_c
  
  
  Products:   
  
      
   pi\_m,   h\_m  
  
  Kinetic mechanism:   A1CSP   
  
  


---

  
  
63      **PPP**:  
  
  
          ppi\_c    <-->    2\*pi\_c    +     2\*h\_c     
  
  
  Substrates:   
  
      
   ppi\_c
  
  
  Products:   
  
      
   pi\_c,   h\_c  
  
  Kinetic mechanism:   CHEM2   
  
  


---

  
  
64      **NDR**:  
  
  
          nadp\_c   +    2\*feri\_m    <-->    nadph\_c    +     2\*fero\_m     
  
  
  Substrates:   
  
      
   nadp\_c,   feri\_m
  
  
  Products:   
  
      
   nadph\_c,   fero\_m  
  
  Kinetic mechanism:   A2i   
  
  


---

  
  
65      **NH4t**:  
  
  
        <-->    nh4\_c     
  
  
  Substrates:   
  
      
   N/A
  
  
  Products:   
  
      
   nh4\_c  
  
  Kinetic mechanism:   A1   
  
  


---

  
  
66      **SO4t**:  
  
  
        <-->    so4\_c    +     h\_c     
  
  
  Substrates:   
  
      
   N/A
  
  
  Products:   
  
      
   so4\_c,   h\_c  
  
  Kinetic mechanism:   A1CSP   
  
  


---

  
  
67      **MLPIT**:  
  
  
          pi\_c   +    mal\_m    <-->    pi\_m    +     mal\_c     
  
  
  Substrates:   
  
      
   pi\_c,   mal\_m
  
  
  Products:   
  
      
   pi\_m,   mal\_c  
  
  Kinetic mechanism:   A2   
  
  


---

  
  
68      **MDHc**:  
  
  
          nad\_c   +    mal\_c    <-->    oaa\_c    +     h\_c    +     nadh\_c     
  
  
  Substrates:   
  
      
   nad\_c,   mal\_c
  
  
  Products:   
  
      
   oaa\_c,   h\_c,   nadh\_c  
  
  Kinetic mechanism:   A2CSP   
  
  


---

  
  
69      **CITc**:  
  
  
          h\_c   +    coa\_c   +    cit\_c    <-->    oaa\_c    +     accoa\_c     
  
  
  Substrates:   
  
      
   h\_c,   coa\_c,   cit\_c
  
  
  Products:   
  
      
   oaa\_c,   accoa\_c  
  
  Kinetic mechanism:   A2CSP   
  
  


---

  
  
70      **ACOc**:  
  
  
          cit\_c    <-->    icit\_c     
  
  
  Substrates:   
  
      
   cit\_c
  
  
  Products:   
  
      
   icit\_c  
  
  Kinetic mechanism:   A1   
  
  


---

  
  
71      **ICL**:  
  
  
          icit\_c    <-->    suc\_c    +     glyx\_c     
  
  
  Substrates:   
  
      
   icit\_c
  
  
  Products:   
  
      
   suc\_c,   glyx\_c  
  
  Kinetic mechanism:   RHUB   
  
  


---

  
  
72      **MLS**:  
  
  
          accoa\_c   +    glyx\_c    <-->    h\_c    +     coa\_c    +     mal\_c     
  
  
  Substrates:   
  
      
   accoa\_c,   glyx\_c
  
  
  Products:   
  
      
   h\_c,   coa\_c,   mal\_c  
  
  Kinetic mechanism:   A2CSP   
  
  


---

  
  
73      **XTR**:  
  
  
        <-->    xyl\_c     
  
  
  Substrates:   
  
      
   N/A
  
  
  Products:   
  
      
   xyl\_c  
  
  Kinetic mechanism:   A1   
  
  


---

  
  
74      **XDH**:  
  
  
          nad\_c   +    xlt\_c    <-->    h\_c    +     nadh\_c    +     xyll\_c     
  
  
  Substrates:   
  
      
   nad\_c,   xlt\_c
  
  
  Products:   
  
      
   h\_c,   nadh\_c,   xyll\_c  
  
  Kinetic mechanism:   A2CSP   
  
  


---

  
  
75      **XRI**:  
  
  
          h\_c   +    nadh\_c   +    xyl\_c    <-->    nad\_c    +     xlt\_c     
  
  
  Substrates:   
  
      
   h\_c,   nadh\_c,   xyl\_c
  
  
  Products:   
  
      
   nad\_c,   xlt\_c  
  
  Kinetic mechanism:   A2CSP   
  
  


---

  
  
76      **XRII**:  
  
  
          nadph\_c   +    h\_c   +    xyl\_c    <-->    nadp\_c    +     xlt\_c     
  
  
  Substrates:   
  
      
   nadph\_c,   h\_c,   xyl\_c
  
  
  Products:   
  
      
   nadp\_c,   xlt\_c  
  
  Kinetic mechanism:   A2CSP   
  
  


---

  
  
77      **XK**:  
  
  
          atp\_c   +    xyll\_c    <-->    adp\_c    +     x5p\_c     
  
  
  Substrates:   
  
      
   atp\_c,   xyll\_c
  
  
  Products:   
  
      
   adp\_c,   x5p\_c  
  
  Kinetic mechanism:   A2CSP   
  
  


---

  
  
78      **XLT**:  
  
  
          xlt\_c    <-->   
  
  
  Substrates:   
  
      
   xlt\_c
  
  
  Products:   
  
      
   N/A  
  
  Kinetic mechanism:   A1   
  
  


---

  
  
79      **D\_LACt2r**:  
  
  
          h\_c   +    lac\_c    <-->   
  
  
  Substrates:   
  
      
   h\_c,   lac\_c
  
  
  Products:   
  
      
   N/A  
  
  Kinetic mechanism:   A1CSP   
  
  


---

  
  
80      **D\_LACm2r**:  
  
  
          h\_m   +    lac\_m    <-->    h\_c    +     lac\_c     
  
  
  Substrates:   
  
      
   h\_m,   lac\_m
  
  
  Products:   
  
      
   h\_c,   lac\_c  
  
  Kinetic mechanism:   A1CSP   
  
  


---

  
  
81      **D\_LAC**:  
  
  
          pyr\_m   +    2\*h\_m   +    2\*fero\_m    <-->    2\*feri\_m    +     lac\_m     
  
  
  Substrates:   
  
      
   pyr\_m,   h\_m,   fero\_m
  
  
  Products:   
  
      
   feri\_m,   lac\_m  
  
  Kinetic mechanism:   A2CSPL   
  
  


---

  
  
82      **SUCCt2r**:  
  
  
          h\_c   +    suc\_c    <-->   
  
  
  Substrates:   
  
      
   h\_c,   suc\_c
  
  
  Products:   
  
      
   N/A  
  
  Kinetic mechanism:   A1CSP   
  
  


---

  
  
83      **MALt2r**:  
  
  
          h\_c   +    mal\_c    <-->   
  
  
  Substrates:   
  
      
   h\_c,   mal\_c
  
  
  Products:   
  
      
   N/A  
  
  Kinetic mechanism:   A1CSP   
  
  


---

  
  
84      **CITt2m**:  
  
  
          mal\_c   +    cit\_m    <-->    mal\_m    +     cit\_c     
  
  
  Substrates:   
  
      
   mal\_c,   cit\_m
  
  
  Products:   
  
      
   mal\_m,   cit\_c  
  
  Kinetic mechanism:   A2CSP   
  
  


---

  
  
