# Supplementary material for: A design–build–test cycle using modeling and experiments reveals interdependencies between upper glycolysis and xylose uptake in recombinant S. cerevisiae and improves predictive capabilities of large-scale kinetic models
Source: Biotechnol Biofuels. 2017 Jun 26;10:166. doi: 10.1186/s13068-017-0838-5 (PMC5485749; doi:10.1186/s13068-017-0838-5)
Supplement: Supplementary file 8 — Additional file 8. Flux Variability Analysis (FVA): (i) obtained ranges of the fluxes of reactions involved in the cytosolic ATP and NADPH metabolism for 4 different values of NDR flux; and (ii) scatter plots of the fluxes of reactions involved in the cytosolic ATP and NADPH metabolism for 4 different values of NDR flux. [file 13068_2017_838_MOESM8_ESM.doc]

We performed Flux Variability Analysis (FVA) and we have found that an increase in NDR reduces allowable ranges for a number of fluxes in the network, primarily the ones that are related to cytosolic NADPH and ATP metabolism. We performed the sampling of the metabolite fluxes to identify couplings of fluxes in these two parts of metabolism.

**Table 1:** FVA of reactions involved in the cytosolic NAPDH metabolism. We have fixed flux of NDR to four different tight ranges ranging from [-0.016,-0.0158] to [-0.48,-0.47] and performed FVA.


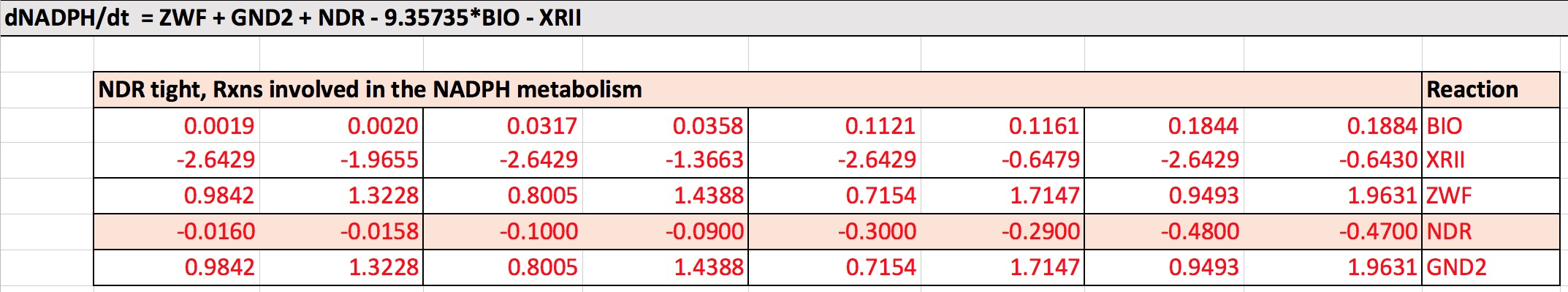


| 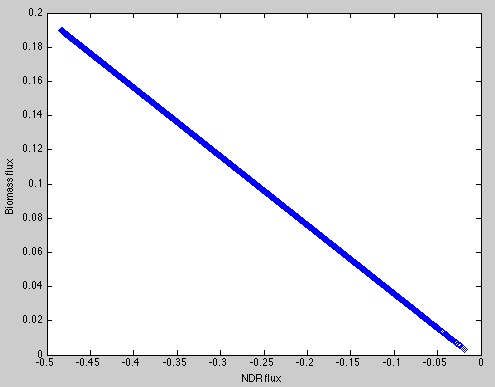 | 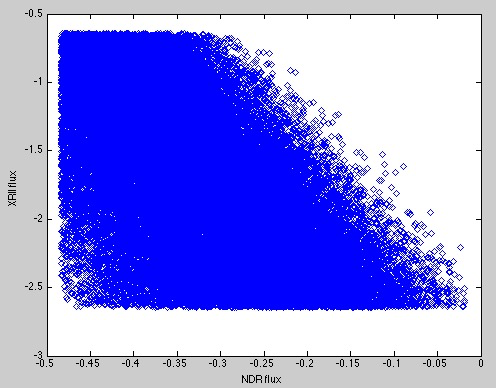 |
| --- | --- |
| 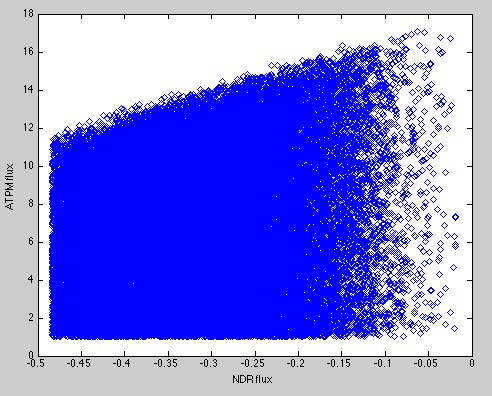 | 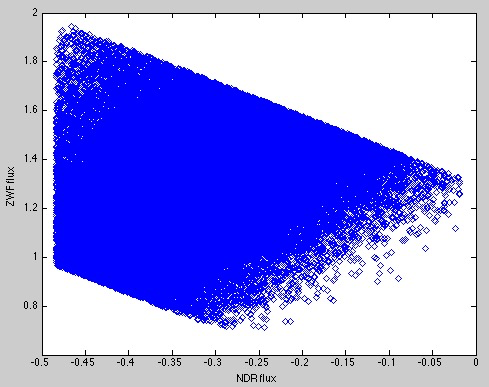 |

**Figure 1:** Sampling of thermodynamically feasible flux values. Correlations between NDR flux and growth (left upper panel), XRII (right upper panel), ATPM (left lower panel) and ZWR (right lower panel).

**Table 2:** FVA of reactions involved in the cytosolic ATP metabolism. We have fixed flux of NDR to four different tight ranges ranging from [-0.016,-0.0158] to [-0.48,-0.47] and performed FVA.


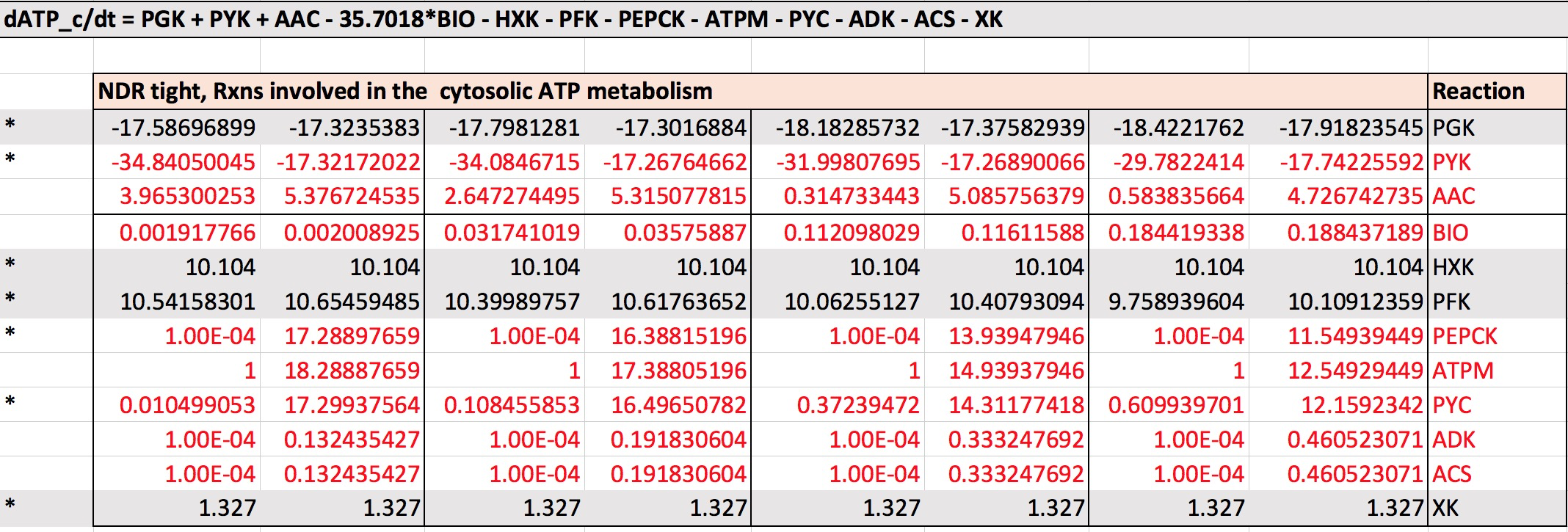


| 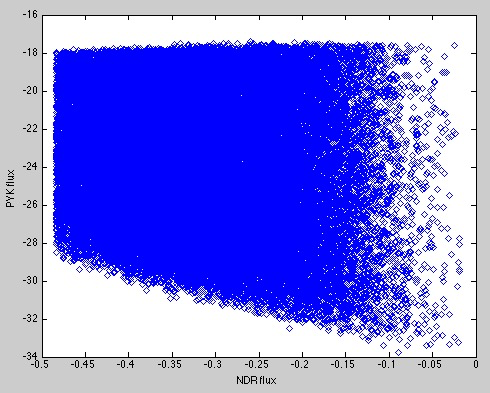 | 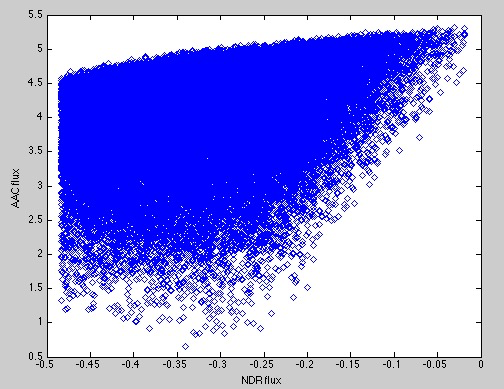 |
| --- | --- |
| 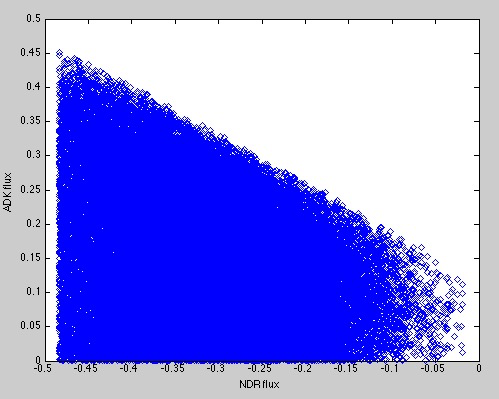 | 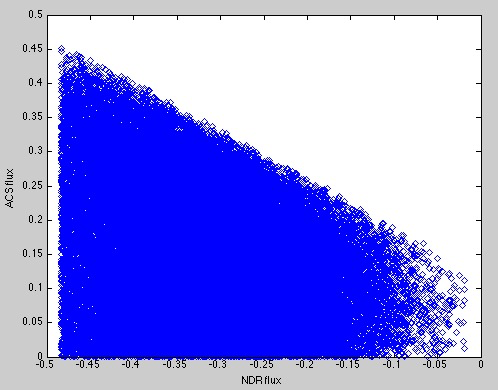 |

**Figure 2:** Sampling of thermodynamically feasible flux values. Correlations between NDR flux and PYK (left upper panel), AAC (right upper panel), ADK (left lower panel) and ACS (right lower panel).

| 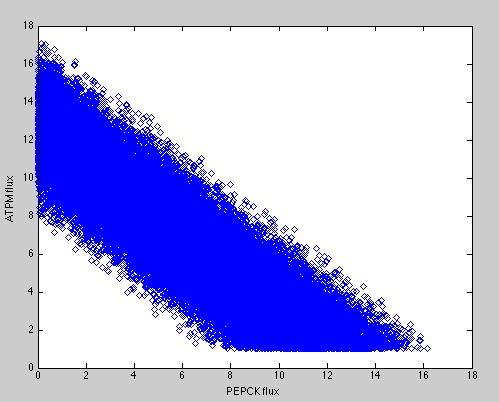 | 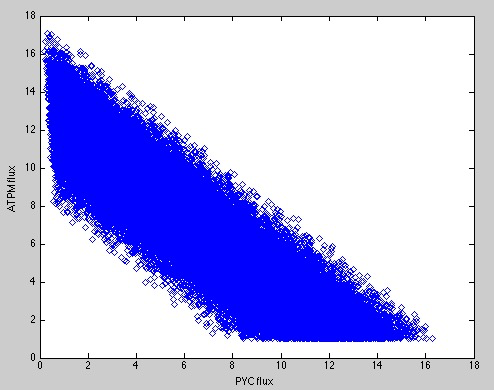 |
| --- | --- |
| 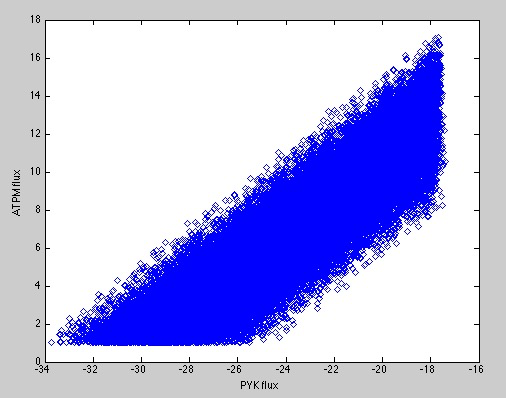 | 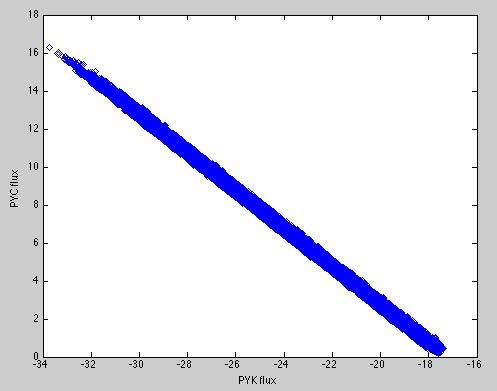 |
| 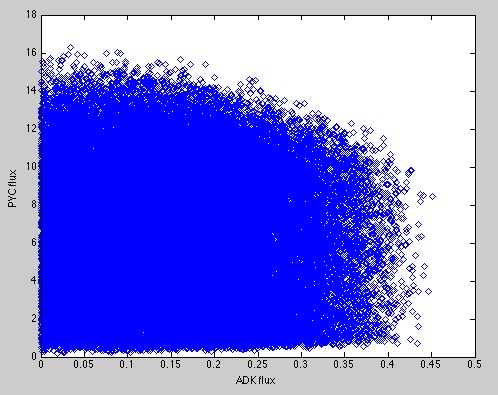 | 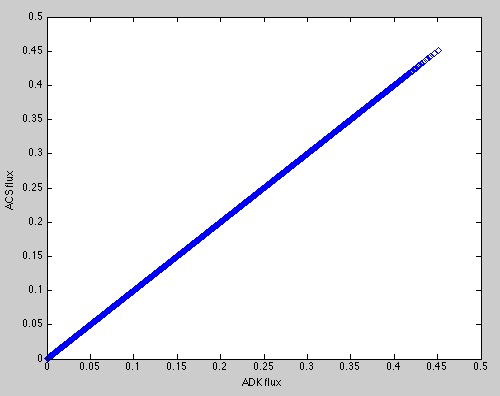 |

**Figure 3:** Sampling of thermodynamically feasible flux values. Correlations between PEPCK and ATPM (left upper panel), PYC and ATPM (right upper panel), PYK and ATPM (left middle panel), PYK and PYC (right middle panel), ADK and PYC (left lower panel) and ADK and ACS (right lower panel).
