# Supplementary material for: A design–build–test cycle using modeling and experiments reveals interdependencies between upper glycolysis and xylose uptake in recombinant S. cerevisiae and improves predictive capabilities of large-scale kinetic models
Source: Biotechnol Biofuels. 2017 Jun 26;10:166. doi: 10.1186/s13068-017-0838-5 (PMC5485749; doi:10.1186/s13068-017-0838-5)
Supplement: Supplementary file 9 — Additional file 9. Estimated parameter values of the kinetic model used to smooth the measurement data together with the raw data (dots) and fitted model outputs (lines) for the cultivation of the VTT C-10880 and HXK2 deletion strains. Correlation parameters for the measurement data and simulated values are also provided. [file 13068_2017_838_MOESM9_ESM.docx]

**Additional File 5**

The ODE-model simulating the cutlivations was set up according to the Materials&Methods section. The parameters values identified for the two strains are summarized in Table 1. Raw measurement data in comparision with the simulated data is shown in Figure 1 for strain VTT C-10880, and in Figure 2 and Figure 3 for the two replicates *HXK2* deletion strain cutlivations. Correlation parameters for the measurement data and simulated values are given in Table *2*.

Table 1: Parameters of the ode-model for smoothing the experimental cultivation data.

| related to | parameter description | parameter | strain  VTT C-10880 | *HXK2* deletion strain |
| --- | --- | --- | --- | --- |
|  |  |  |  |  |
| substrates | max glucose uptake per g_cdw_ | $Y_{glu}$ | 6.38 | 4.00 |
|  | max xylose uptake per g_cdw_ | $Y_{xyl}$ | 1.76 | 3.84 |
|  | limitation glucose | $k_{m,glu}$ | 9.00 | 10.00 |
|  | limitation xylose | $k_{m,xyl}$ | 145 | 250 |
|  | lag glucose uptake | $\tau_{glu}$ | 1.50 | 3.50 |
|  | lag xylose uptake | $\tau_{xyl}$ | 1.50 | 3.50 |
|  |  |  |  |  |
| main products | yield xylitol from xylose | $Y_{xyl,xli}$ | 0.48 | 0.50 |
|  | yield glycerol from glucose | $Y_{glu,gol}$ | 0.13 | 0.12 |
|  | yield glycerol from xylose | $Y_{xyl,gol}$ | 0.034 | 0.015 |
|  | yield acetate from glucose | $Y_{glu,ace}$ | 0.014 | 0.0029 |
|  | yield acetate from xylose | $Y_{xyl,ace}$ | 0.012 | 0.0079 |
|  |  |  |  |  |
| ethanol | yield ethanol from glucose | $Y_{glu,eth}$ | 0.34 | 0.45 |
|  | yield ethanol from xylose | $Y_{xyl,eth}$ | 0.17 | 0.08 |
|  |  |  |  |  |
| co2 | yield co2 from glucose | $Y_{xyl,co2}$ | 0.42 | 0.44 |
|  | yield co2 from xylose | $Y_{xyl,co2}$ | 0.29 | 0.25 |
|  |  |  |  |  |
| growth | growth on glucose | $\mu_{glu}$ | 0.06 | 0.035 |
|  | growth on xylose | $\mu_{xyl}$ | 0.01 | 0.008 |
|  |  |  |  |  |


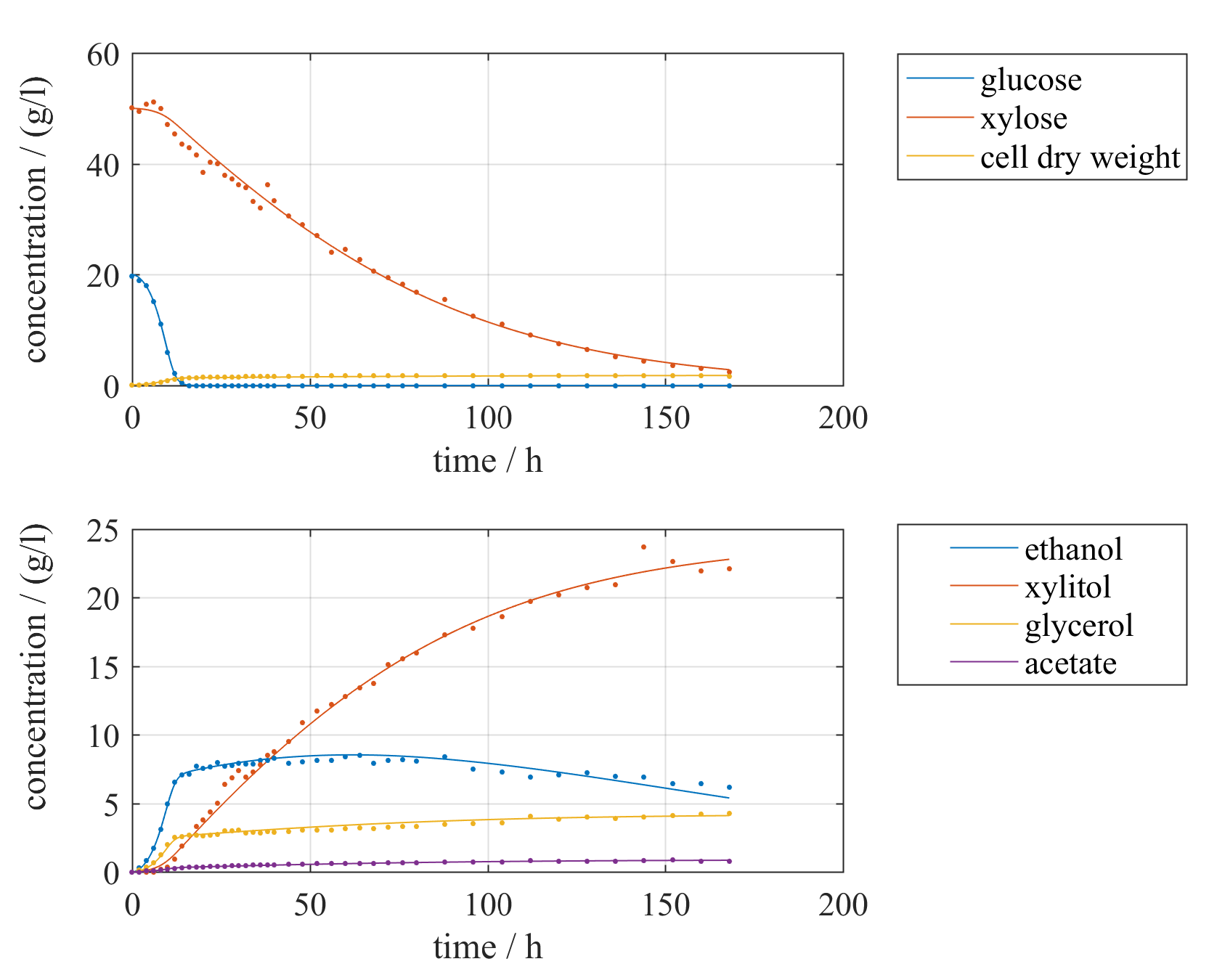


Figure 1: Measurements (dots) and fitted model (lines) for the cultivation with strain VTT C-10880.


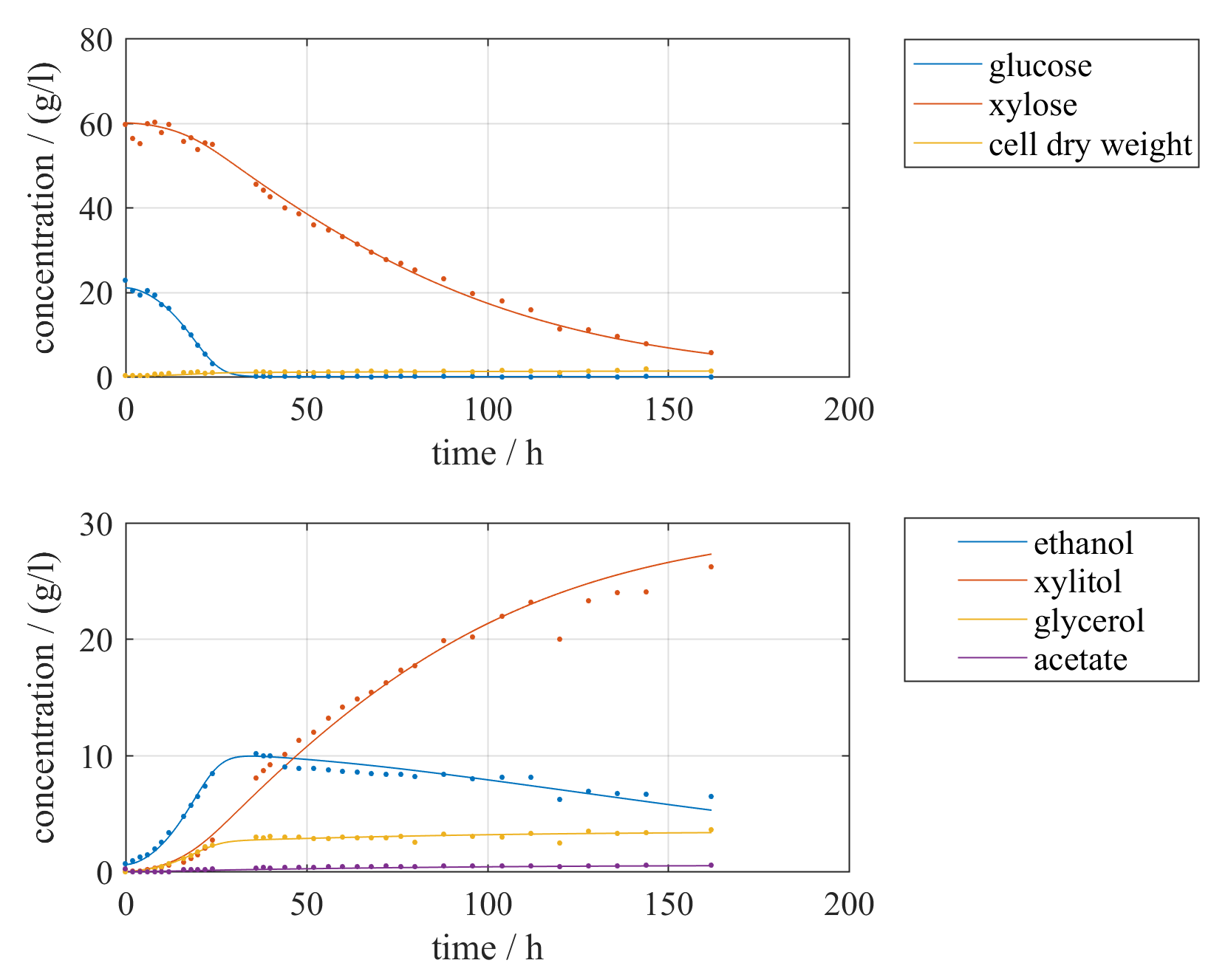


Figure 2: Measurements (dots) and fitted model (lines) for the cultivation with the HXK2 deletion strain (cultivation1/2).


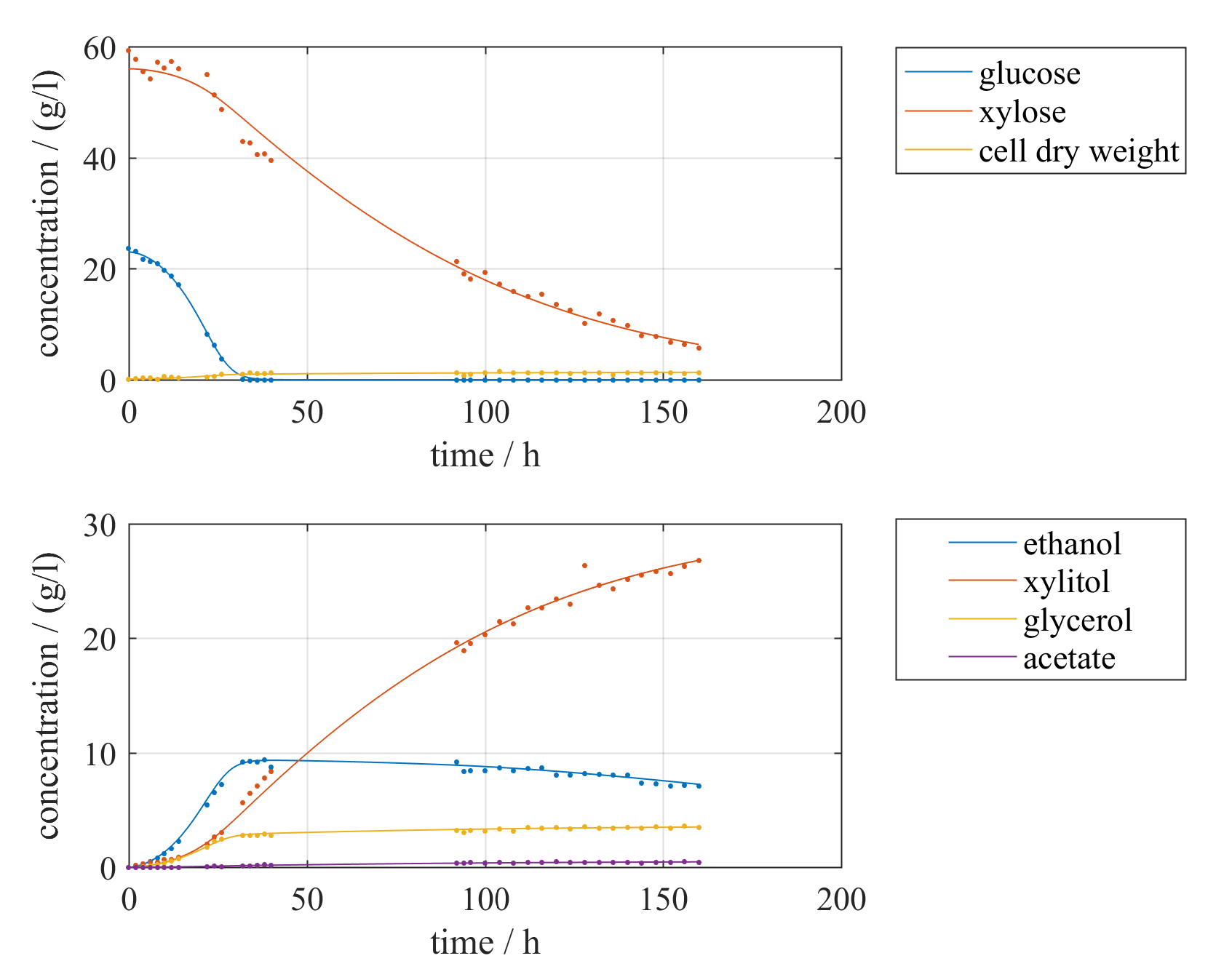


Figure 3: Measurements (dots) and fitted model (lines) for the cultivation with the HXK2 deletion strain (cultivation2/2).

Table 2: Correlation coefficiets, experimental data vs simulation data

|  | strain  VTT C-10880 | *HXK2* deletion strain (1/2) | *HXK2* deletion strain (2/2) |
| --- | --- | --- | --- |
| glucose | 0.9999 | 0.9985 | 0.9996 |
| xylose | 0.9967 | 0.9975 | 0.9957 |
| cell dry weight | 0.9863 | 0.8683 | 0.9122 |
| ethanol | 0.9909 | 0.9883 | 0.9975 |
| xylitol | 0.9976 | 0.9939 | 0.9985 |
| glycerol | 0.9894 | 0.9846 | 0.9978 |
| acetate | 0.9925 | 0.9504 | 0.9818 |
