# Supplementary material for: A design–build–test cycle using modeling and experiments reveals interdependencies between upper glycolysis and xylose uptake in recombinant S. cerevisiae and improves predictive capabilities of large-scale kinetic models
Source: Biotechnol Biofuels. 2017 Jun 26;10:166. doi: 10.1186/s13068-017-0838-5 (PMC5485749; doi:10.1186/s13068-017-0838-5)
Supplement: Supplementary file 10 — Additional file 10. Rate expressions for used kinetic mechanisms and the expressions for the corresponding metabolite elasticities. [file 13068_2017_838_MOESM10_ESM.docx]

# Rate expressions for kinetic mechanisms and metabolite elasticities

**Kinetic mechanism:** A1

This mechanism follows uni-uni reversible Michaelis-Menten kinetics,

$v_{f}=v_{\max,f}\frac{s}{1+s+p}$, $v_{b}=v_{\max,b}\frac{p}{1+s+p}$.

where $s$ is the scaled concentrations of the substrate, and $p$ is the scaled concentrations of the product. The elasticities have the forms

$\varepsilon_{f,s}=\frac{1+p}{1+s+p}$,

$\varepsilon_{f,p}=\frac{-p}{1+s+p}$,

$\varepsilon_{b,s}=\frac{-s}{1+s+p}$,

$\varepsilon_{b,p}=\frac{1+s}{1+s+p}$.

**Kinetic mechanism:** A1CSP

The elasticities for the substrate, and for the product have the same expressions as in the kinetic mechanism A1. The elasticities for the compensated species are modeled in the following way:

$$\varepsilon_{f, c1}=1$$

$$\varepsilon_{b, c1}=0$$

$$\varepsilon_{f, c2}=0$$

$$\varepsilon_{b, c2}=1$$

**In general, the suffix CSnPm in the name of the kinetic mechanism indicates that n species are compensated substrate side and m species are compensated product side.**

**Kinetic mechanism:** A1CSPh13

The elasticities for the substrate, and for the product have the same expressions as in the kinetic mechanism A1. The elasticities for the compensated species are modeled in the following way:

$$\varepsilon_{f, c1}=1$$

$$\varepsilon_{b, c1}=0$$

$$\varepsilon_{f, c2}=0$$

$$\varepsilon_{b, c2}=3$$

**Kinetic mechanism:** A1CS2P2

The elasticities for the substrate, and for the product have the same expressions as in the kinetic mechanism A1. The elasticities for the two compensated substrates c1 and c2 and for the two compensated products c3 and c4 are:

$$\varepsilon_{f, c1}=1$$

$$\varepsilon_{b, c1}=0$$

$$\varepsilon_{f, c2}=1$$

$$\varepsilon_{b, c2}=0$$

$$\varepsilon_{f, c3}=0$$

$$\varepsilon_{b, c3}=1$$

$$\varepsilon_{f, c4}=0$$

$$\varepsilon_{b, c4}=1$$

**Kinetic mechanism:** A1CSP2h112

The elasticities for the substrate, and for the product have the same expressions as in the kinetic mechanism A1. The elasticities for the compensated substrate c1 and for the two compensated products c2 and c3 are:

$$\varepsilon_{f, c1}=1$$

$$\varepsilon_{b, c1}=0$$

$$\varepsilon_{f, c2}=0$$

$$\varepsilon_{b, c2}=1$$

$$\varepsilon_{f, c3}=0$$

$$\varepsilon_{b, c3}=2$$

**Kinetic mechanism:** A1CSP3

The elasticities for the substrate, and for the product have the same expressions as in the kinetic mechanism A1. The elasticities for the compensated substrate c1 and for the three compensated products c2, c3 and c4 are:

$$\varepsilon_{f, c1}=1$$

$$\varepsilon_{b, c1}=0$$

$$\varepsilon_{f, c2}=0$$

$$\varepsilon_{b, c2}=1$$

$$\varepsilon_{f, c3}=0$$

$$\varepsilon_{b, c3}=1$$

$$\varepsilon_{f, c4}=0$$

$$\varepsilon_{b, c4}=1$$

**Kinetic mechanism:** A1CP2i

The elasticities for the substrate and for the product have the same expressions as in the kinetic mechanism A1. The elasticities for the two compensated products c1 and c2 are:

$$\varepsilon_{f, c1}=0$$

$$\varepsilon_{b, c1}=1$$

$$\varepsilon_{f, c2}=0$$

$$\varepsilon_{b, c2}=1$$

The small letter “i” in the name of the kinetic mechanism denotes that this reaction is *kinetically* favorable in the forward direction. To model this effect we have imposed that the probability of the occupancy of the active site by the products is very low.

**Kinetic mechanism:** A2

Generalized reversible Hill with two substrates and two products and with the coefficient $h=1$:

$v_{f}=v_{\max,f}\frac{s_{1}s_{2}}{\left(1+s_{1}+p_{1}\right)\left(1+b_{2}+p_{2}\right)}$,

$v_{b}=v_{\max,b}\frac{p_{1}p_{2}}{\left(1+s_{1}+p_{1}\right)\left(1+s_{2}+p_{2}\right)}$.

where $s_{1}$ and $s_{2}$ are the scaled concentrations of substrates, while $p_{1}$ and $p_{2}$ are the scaled concentrations of products. The elasticities can be expressed as

$\varepsilon_{f,s_{1}}=\frac{1+p_{1}}{1+s_{1}+p_{1}}$,

$\varepsilon_{f,s_{2}}=\frac{1+p_{2}}{1+s_{2}+p_{2}}$,

$\varepsilon_{f,p_{1}}=\frac{-p_{1}}{1+s_{1}+p_{1}}$,

$\varepsilon_{f,p_{2}}=\frac{-p_{2}}{1+s_{2}+p_{2}}$,

$\varepsilon_{b,s_{1}}=\frac{-s_{1}}{1+s_{1}+p_{1}}$,

$\varepsilon_{b,s_{2}}=\frac{-s_{2}}{1+s_{2}+p_{2}}$,

$\varepsilon_{b,p_{1}}=\frac{1+s_{1}}{1+s_{1}+p_{1}}$,

$\varepsilon_{b,p_{2}}=\frac{1+s_{2}}{1+s_{2}+p_{2}}$.

**Kinetic mechanism:** A2CSP

The elasticities for the substrates and for the products have the same expressions as in the kinetic mechanism A2. The elasticities for the compensated species are modeled in the following way:

$$\varepsilon_{f, c1}=1$$

$$\varepsilon_{b, c1}=0$$

$$\varepsilon_{f, c2}=0$$

$$\varepsilon_{b, c2}=1$$

**Kinetic mechanism:** A2CSPh22

The elasticities for the substrates, and for the products have the same expressions as in the kinetic mechanism A2. The elasticities for the compensated species are modeled in the following way:

$$\varepsilon_{f, c1}=2$$

$$\varepsilon_{b, c1}=0$$

$$\varepsilon_{f, c2}=0$$

$$\varepsilon_{b, c2}=2$$

**Kinetic mechanism:** A2CSPh43

The elasticities for the substrates, and for the products have the same expressions as in the kinetic mechanism A2. The elasticities for the compensated species are modeled in the following way:

$$\varepsilon_{f, c1}=4$$

$$\varepsilon_{b, c1}=0$$

$$\varepsilon_{f, c2}=0$$

$$\varepsilon_{b, c2}=3$$

**Kinetic mechanism:** A2CS2P2

The elasticities for the substrates, and for the products have the same expressions as in the kinetic mechanism A2. The elasticities for the two compensated substrates c1 and c2 and for the two compensated products c3 and c4 are:

$$\varepsilon_{f, c1}=1$$

$$\varepsilon_{b, c1}=0$$

$$\varepsilon_{f, c2}=1$$

$$\varepsilon_{b, c2}=0$$

$$\varepsilon_{f, c3}=0$$

$$\varepsilon_{b, c3}=1$$

$$\varepsilon_{f, c4}=0$$

$$\varepsilon_{b, c4}=1$$

**Kinetic mechanism:** A6CS

It is the ordered *bi-bi* kinetics, with cofactor binding first, and with one compensated substrate

,

,

where $s_{1}$ and $s'_{1}$ are the scaled concentration of substrate 1 with respect to two different enzymatic constants, $s_{2}$ and $s'_{2}$ are the scaled concentration of substrate 2 with respect to two different enzymatic constants, $p_{1}$ and $p'_{1}$ are the scaled concentration of product 1 with respect to two different enzymatic constants, $p_{2}$ and $p'_{2}$ are the scaled concentration of product 2 with respect to two different enzymatic constants. The elasticities can be expressed as

$\varepsilon_{f,S1}=\frac{1+s'_{2}+p'_{1}+p_{2}+s'_{2}p_{2}+p_{1}p_{2}+s_{2}p_{1}p'_{2}}{1+s_{1}+s'_{2}+p'_{1}+p_{2}+s_{1}s_{2}+s_{1}p'_{1}+s'_{2}p_{2}+p_{1}p_{2}+s'_{1}s_{2}p_{1}+s_{2}p_{1}p'_{2}}$,

$\varepsilon_{f,S2}=\frac{1+s_{1}+p'_{1}+p_{2}+s_{1}p'_{1}+p_{1}p_{2}}{1+s_{1}+s'_{2}+p'_{1}+p_{2}+s_{1}s_{2}+s_{1}p'_{1}+s'_{2}p_{2}+p_{1}p_{2}+s'_{1}s_{2}p_{1}+s_{2}p_{1}p'_{2}}$,

$\varepsilon_{f,P1}=\frac{-\left(p'_{1}+s_{1}p'_{1}+p_{1}p_{2}+s'_{1}s_{2}p_{1}+s_{2}p_{1}p'_{2}\right)}{1+s_{1}+s'_{2}+p'_{1}+p_{2}+s_{1}s_{2}+s_{1}p'_{1}+s'_{2}p_{2}+p_{1}p_{2}+s'_{1}s_{2}p_{1}+s_{2}p_{1}p'_{2}}$,

$\varepsilon_{f,P2}=\frac{-\left(p_{2}+s'_{2}p_{2}+p_{1}p_{2}+s_{2}p_{1}p'\right)_{2}}{1+s_{1}+s'_{2}+p'_{1}+p_{2}+s_{1}s_{2}+s_{1}p'_{1}+s'_{2}p_{2}+p_{1}p_{2}+s'_{1}s_{2}p_{1}+s_{2}p_{1}p'_{2}}$,

$\varepsilon_{b,S1}=\frac{-\left(s_{1}+s_{1}s_{2}+s_{1}p'_{1}+s'_{1}s_{2}p_{1}\right)}{1+s_{1}+s'_{2}+p'_{1}+p_{2}+s_{1}s_{2}+s_{1}p'_{1}+s'_{2}p_{2}+p_{1}p_{2}+s'_{1}s_{2}p_{1}+s_{2}p_{1}p'_{2}}$,

$\varepsilon_{b,S2}=\frac{-\left(s'_{2}+s_{1}s_{2}+s'_{2}p_{2}+s'_{1}s_{2}p_{1}+s_{2}p_{1}p'\right)}{1+s_{1}+s'_{2}+p'_{1}+p_{2}+s_{1}s_{2}+s_{1}p'_{1}+s'_{2}p_{2}+p_{1}p_{2}+s'_{1}s_{2}p_{1}+s_{2}p_{1}p'_{2}}$,

$\varepsilon_{b,P1}=\frac{1+s_{1}+s'_{2}+p_{2}+s_{1}s_{2}+s'_{2}p_{2}}{1+s_{1}+s'_{2}+p'_{1}+p_{2}+s_{1}s_{2}+s_{1}p'_{1}+s'_{2}p_{2}+p_{1}p_{2}+s'_{1}s_{2}p_{1}+s_{2}p_{1}p'_{2}}$,

$\varepsilon_{b,P2}=\frac{1+s_{1}+s'_{2}+p'_{1}+s_{1}s_{2}+s_{1}p'_{1}+s'_{1}s_{2}p_{1}}{1+s_{1}+s'_{2}+p'_{1}+p_{2}+s_{1}s_{2}+s_{1}p'_{1}+s'_{2}p_{2}+p_{1}p_{2}+s'_{1}s_{2}p_{1}+s_{2}p_{1}p'_{2}}$.

We generate random independent samples of scaled metabolite concentrations, $s_{1}$, $s'_{1}$, $s_{2}$, $s'_{2}$, $p_{1}$, $p'_{1}$, $p_{2}$, $p'_{2}$. These samples are used to calculate the elasticities.

The elasticities for the compensated substrate c1 are modeled in the following way:

$$\varepsilon_{f, c1}=1$$

$$\varepsilon_{b, c1}=0$$

**Kinetic mechanism:** A99

Generalized reversible Hill with two substrates and two products, and with the allosteric regulation:

PEP acts as external inhibitor and ADP as activator.

We rename the concentrations of F6P, ATP, F1,6bP, ADP and PEP as $S_{1}$, $S_{2}$, $P_{1}$, $P_{2}$ and $M$, respectively. The reversible Hill rate law for the PFK in the case of independent and dependent modifiers ($v_{i,PFK}$ and $v_{d,PFK}$, respectively) can thus be written as:

$v_{i,PFK}=\frac{V_{f,max}\widetilde{S_{1}}\widetilde{S_{2}}W_{1}W_{2}}{Q_{1}T_{1}+Q_{2}T_{2}T_{3}+T_{4}}\left(1-\Gamma\right)$,

$v_{d,PFK}=\frac{V_{f,max}\widetilde{S_{1}}\widetilde{S_{2}}W_{1}W_{2}}{Z_{1}+Z_{2}T_{3}+T_{4}}\left(1-\Gamma\right)$,

where

$W_{1}=(\widetilde{S_{1}}+\widetilde{P_{1}})^{3}$,

$W_{2}=(\widetilde{S_{2}}+\widetilde{P_{2}})^{3}$,

$Q_{1}=\frac{1+\tilde{M}^{4}}{1+m^{2}\tilde{M}^{4}}$,

$T_{1}=\frac{1+\widetilde{P_{2}}^{4}}{1+p^{2}\widetilde{P_{2}}^{4}}$,

$Q_{2}=\frac{1+m\tilde{M}^{4}}{1+m^{2}\tilde{M}^{4}}$,

$T_{2}=\frac{1+p\widetilde{P_{2}}^{4}}{1+p^{2}\widetilde{P_{2}}^{4}}$,

$T_{3}=(\widetilde{S_{1}}+\widetilde{P_{1}})^{4}+(\widetilde{S_{2}}+\widetilde{P_{2}})^{4}$,

$T_{4}=(\widetilde{S_{1}}+\widetilde{P_{1}})^{4}(\widetilde{S_{2}}+\widetilde{P_{2}})^{4}$,

$Z_{1}=\frac{1+\tilde{M}^{4}+\widetilde{P_{2}}^{4}}{1+m^{2}\tilde{M}^{4}+p^{2}\widetilde{P_{2}}^{4}}$,

$Z_{2}=\frac{1+m\tilde{M}^{4}+p\widetilde{P_{2}}^{4}}{1+m^{2}\tilde{M}^{4}+p^{2}\widetilde{P_{2}}^{4}}$.

The effects of inhibition by PEP and activation by ADP are given by the two parameters $0<m<1$ and $p>1$.

Given that $D_{i}=Q_{1}T_{1}+Q_{2}T_{2}T_{3}+T_{4}$, the expressions for the irreversible forward metabolite elasticities of PFK in the case of independent modifiers are the following:

$\varepsilon_{S_{1}}^{i}=1+\frac{3\widetilde{S_{1}}}{\widetilde{S_{1}}+\widetilde{P_{1}}}-\frac{4\widetilde{S_{1}}W_{1}(W_{2}\widetilde{S_{2}}+W_{2}\widetilde{P_{2}}+Q_{2}T_{2})}{D_{i}}$,

$\varepsilon_{S_{2}}^{i}\text{ }=1+\frac{3\widetilde{S_{2}}}{\widetilde{S_{2}}+\widetilde{P_{2}}}-\frac{4\widetilde{S_{2}}W_{2}(W_{1}\widetilde{S_{1}}+W_{1}\widetilde{P_{1}}+Q_{2}T_{2})}{D_{i}}$,

$\varepsilon_{P_{1}}^{i}=\frac{3\widetilde{P_{1}}}{\widetilde{S_{1}}+\widetilde{P_{1}}}-\frac{4\widetilde{P_{1}}W_{1}(W_{2}\widetilde{S_{2}}+W_{2}\widetilde{P_{2}}+Q_{2}T_{2})}{D_{i}}$,

$\varepsilon_{P_{2}}^{i}=\frac{3\widetilde{P_{2}}}{\widetilde{S_{2}}+\widetilde{P_{2}}}-\frac{4\widetilde{P_{2}}}{D_{i}}\left[\frac{\widetilde{P_{2}}^{3}(Q_{1}(1-p^{2}T_{1})+pQ_{2}T_{3}(1-pT_{2}))}{1+p^{2}\widetilde{P_{2}}^{4}}+Q_{2}T_{2}W_{2}+\frac{T_{4}}{\widetilde{S_{2}}+\widetilde{P_{2}}}\right]$,

$\varepsilon_{M}^{i}=-\frac{4\tilde{M}^{4}}{D_{i}}\left[\frac{T_{1}(1-m^{2}Q_{1})+T_{2}T_{3}m(1-mQ_{2})}{1+m^{2}\tilde{M}^{4}}\right]$.

Given that $D_{d}=Z_{1}+Z_{2}T_{3}+T_{4}$, the expressions for the irreversible forward metabolite elasticities of PFK in the case of dependent modifiers are the following:

$\varepsilon_{S_{1}}^{d}=1+\frac{3\widetilde{S_{1}}}{\widetilde{S_{1}}+\widetilde{P_{1}}}-\frac{4\widetilde{S_{1}}W_{1}(W_{2}\widetilde{S_{2}}+W_{2}\widetilde{P_{2}}+Z_{2})}{D_{d}}$,

$\varepsilon_{S_{2}}^{d}=1+\frac{3\widetilde{S_{2}}}{\widetilde{S_{2}}+\widetilde{P_{2}}}-\frac{4\widetilde{S_{2}}W_{2}(W_{1}\widetilde{S_{1}}+W_{1}\widetilde{P_{1}}+Z_{2})}{D_{d}}$,

$\varepsilon_{P_{1}}^{d}=\frac{3\widetilde{P_{1}}}{\widetilde{S_{1}}+\widetilde{P_{1}}}-\frac{4\widetilde{P_{1}}W_{1}(W_{2}\widetilde{S_{2}}+W_{2}\widetilde{P_{2}}+Z_{2})}{D_{d}}$,

$\varepsilon_{P_{2}}^{d}=\frac{3\widetilde{P_{2}}}{\widetilde{S_{2}}+\widetilde{P_{2}}}-\frac{4\widetilde{P_{2}}}{D_{d}}\left[\frac{\widetilde{P_{2}}^{3}(1-p^{2}Z_{1}+pT_{3}-p^{2}T_{3}Z_{2})}{1+m^{2}\tilde{M}^{4}+p^{2}\widetilde{P_{2}}^{4}}+Z_{2}W_{2}+\frac{T_{4}}{\widetilde{S_{2}}+\widetilde{P_{2}}}\right]$,

$\varepsilon_{M}^{d}=-\frac{4\tilde{M}^{4}}{D_{d}}\left[\frac{1-m^{2}Z_{1}+mT_{3}-m^{2}T_{3}Z_{2}}{1+m^{2}\tilde{M}^{4}+p^{2}\widetilde{P_{2}}^{4}}\right]$.

**Kinetic mechanism:** B4

It is the reversible Michaelis-Menten type kinetics as

$v_{f}=v_{\max,f}\frac{mt}{1+2d+m+t}$,

$v_{b}=v_{\max,b}\frac{d^{2}}{1+2d+m+t}$,

where $d$, $m$, and $t$ represent the scaled concentrations of $P_{1}$, $S_{1}$, and $S_{2}$, respectively. Their elasticities are expressed in the following equations:

$\varepsilon_{f,S1}=\frac{1+2d+t}{1+2d+m+t}$,

$\varepsilon_{f,S2}=\frac{1+2d+m}{1+2d+m+t}$,

$\varepsilon_{f,P1}=\frac{-2d}{1+2d+m+t}$,

$\varepsilon_{b,S1}=\frac{-m}{1+2d+m+t}$,

$\varepsilon_{b,S2}=\frac{-t}{1+2d+m+t}$,

$\varepsilon_{b,P1}=\frac{2\left(1+d+m+t\right)}{1+2d+m+t}$.

**Kinetic mechanism:** BTCS

Bi-Ter kinetics mechanism modeled using the convenience kinetics

$v=\frac{v_{\max,f}s_{1}s_{2}-v_{\max,b}p_{1}p_{2}p_{3}}{\left(1+s_{1}\right)\left(1+s_{2}\right)+\left(1+p_{1}\right)\left(1+p_{2}\right)\left(1+p_{3}\right)-1}=\frac{v_{\max,f}s_{1}s_{2}-v_{\max,b}p_{1}p_{2}p_{3}}{D}$.

The elasticities are calculated as

,

$\varepsilon_{f, p_{1}}=\frac{-p_{1}(1+p_{2})(1+p_{3})}{D}$,

$\varepsilon_{f, p_{2}}=\frac{-p_{2}\left(1+p_{1}\right)(1+p_{3})}{D}$,

$$\varepsilon_{f, p_{3}}=\frac{-p_{3}\left(1+p_{1}\right)(1+p_{2})}{D}$$

,

,

$$\varepsilon_{b, p_{1}}=1-\frac{p_{1}(1+p_{2})(1+p_{3})}{D}$$

$\varepsilon_{b, p_{2}}=1-\frac{p_{2}(1+p_{1})(1+p_{3})}{D}$.

.

The elasticities for the compensated substrate c1 are modeled in the following way:

$$\varepsilon_{f, c1}=1$$

$$\varepsilon_{b, c1}=0$$

**Kinetic mechanism:** BTCSP

This kinetic mechanism is like BTCS, but it has one compensated substrate c1 and one compensated product c2. For the compensated species, the elasticities are:

$$\varepsilon_{f, c1}=1$$

$$\varepsilon_{b, c1}=0$$

$$\varepsilon_{f, c2}=0$$

$$\varepsilon_{b, c2}=1$$

**Kinetic mechanism:** CHEM2

The elasticities for this reaction are as follows:

$$\varepsilon_{f, s_{1}}=1$$

$$\varepsilon_{f, p_{1}}=0$$

$$\varepsilon_{f, p_{2}}=0$$

$$\varepsilon_{b, s_{1}}=0$$

$$\varepsilon_{b, p_{1}}=2$$

$$\varepsilon_{b, p_{2}}=2$$

**Kinetic mechanism:** CONVKIN

The general convenience kinetics form is:

The elasticities are provided for general stoichiometry and for any possible coefficient of substrates and products.

**Kinetic mechanism:** RH3CS2P2

Ter-Ter kinetic mechanism modeled by the generalized reversible Hill kinetics.

$v=\frac{v_{max,f}\prod_{i=1}^{3} \frac{s_{i}}{k_{s_{i}}}(1-\Gamma)}{\prod_{i=1}^{3} (1+\frac{s_{i}}{k_{s_{i}}}+\frac{p_{i}}{k_{p_{i}}})}$.

The forward and backward metabolite elasticities are given by:

$\varepsilon_{f,s_{i}}=\frac{1+p_{i}}{1+s_{i}+p_{i}}$,

$\varepsilon_{f,p_{i}}=\frac{-p_{i}}{1+s_{i}+p_{i}}$,

$\varepsilon_{b,s_{i}}=\frac{-s_{i}}{1+s_{i}+p_{i}}$,

$\varepsilon_{b,p_{i}}=\frac{1+s_{i}}{1+s_{i}+p_{i}}$.

In addition, this Kinetic mechanism has two compensated substrates c1 and c2 and two compensated products c3 and c4. For the compensated species, the elasticities are:

$$\varepsilon_{f, c1}=1$$

$$\varepsilon_{b, c1}=0$$

$$\varepsilon_{f, c2}=1$$

$$\varepsilon_{b, c2}=0$$

$$\varepsilon_{f, c3}=0$$

$$\varepsilon_{b, c3}=1$$

$$\varepsilon_{f, c4}=0$$

$$\varepsilon_{b, c4}=1$$

**Kinetic mechanism:** RHUB

We model this uni-bi kinetic mechanism with the reversible Hill kinetic approximation:

$v=v_{\max,f}\frac{s_{1}\left(1-\frac{\Gamma}{K_{eq}}\right)}{1+s_{1}+p_{1}+p_{2}+p_{1}p_{2}}=v_{\max,f}\frac{s_{1}\left(1-\frac{\Gamma}{K_{eq}}\right)}{D}$,

where $s_{1}$ is the scaled concentration of the substrate, $p_{1}$ and $p_{2}$ are the scaled concentrations of first and second product. The elasticities are calculated as

$\varepsilon_{f, s}=1-\frac{s_{1}}{D}$,

$\varepsilon_{f, p_{1}}=\frac{-p_{1}(1+p_{2})}{D}$,

$\varepsilon_{f, p_{2}}=\frac{-p_{2}\left(1+p_{1}\right)}{D}$,

$\varepsilon_{b, s}=\frac{-s_{1}}{D}$,

$\varepsilon_{b, p_{1}}=1-\frac{p_{1}(1+p_{2})}{D}$,

$\varepsilon_{b, p_{2}}=1-\frac{p_{2}(1+p_{1})}{D}$.

**Kinetic mechanism:** RHUBCSP

This kinetic mechanism is like RHUB, but it has one compensated substrate c1 and one compensated product c2, whose elasticities are:

$$\varepsilon_{f, c1}=1$$

$$\varepsilon_{b, c1}=0$$

$$\varepsilon_{f, c2}=0$$

$$\varepsilon_{b, c2}=1$$

**Kinetic mechanism:** RHUBCS2P

This kinetic mechanism is like RHUB, but it has two compensated substrates c1 and c2 and one compensated product c3, whose elasticities are:

$$\varepsilon_{f, c1}=1$$

$$\varepsilon_{b, c1}=0$$

$$\varepsilon_{f, c2}=1$$

$$\varepsilon_{b, c2}=0$$

$$\varepsilon_{f, c3}=0$$

$$\varepsilon_{b, c3}=1$$

**Kinetic mechanism:** RHBU

We model this bi-uni kinetic mechanism with the reversible Hill kinetic approximation:

$v=v_{\max,f}\frac{s_{1}s_{2}\left(1-\frac{\Gamma}{K_{eq}}\right)}{1+s_{1}+p_{1}+s_{2}+s_{1}s_{2}}=v_{\max,f}\frac{s_{1}s_{2}\left(1-\frac{\Gamma}{K_{eq}}\right)}{D}$,

where $s_{1}$ and $s_{2}$ are the scaled concentration of first and second substrate, $p_{1}$ is the scaled concentrations of the product. The elasticities are calculated as

,

,

$\varepsilon_{f, p_{1}}=\frac{-p_{1}}{D}$,

,

,

$\varepsilon_{b, p_{1}}=1-\frac{p_{1}}{D}$.

**Kinetic mechanism:** TBCSP

Ter-bi kinetics mechanism modeled by the convenience kinetics

$v=\frac{v_{\max,f}s_{1}s_{2}s_{3}-v_{\max,b}p_{1}p_{2}}{\left(1+s_{1}\right)\left(1+s_{2}\right)\left(1+s_{3}\right)+\left(1+p_{1}\right)\left(1+p_{2}\right)-1}=\frac{v_{\max,f}s_{1}s_{2}s_{3}-v_{\max,b}p_{1}p_{2}}{D}$,

The elasticities are calculated as

,

$\varepsilon_{f, p_{1}}=\frac{-p_{1}(1+p_{2})}{D}$,

$\varepsilon_{f, p_{2}}=\frac{-p_{2}\left(1+p_{1}\right)}{D}$, ,

,

,

$$\varepsilon_{b, p_{1}}=1-\frac{p_{1}(1+p_{2})}{D}$$

$\varepsilon_{b, p_{2}}=1-\frac{p_{2}(1+p_{1})}{D}$.

The model has one compensated substrate c1 and one compensated product c2, whose elasticities are:

$$\varepsilon_{f, c1}=1$$

$$\varepsilon_{b, c1}=0$$

$$\varepsilon_{f, c2}=0$$

$$\varepsilon_{b, c2}=1$$

**Kinetic mechanism:** TBCS2P2

This mechanism is like TBCSP, but it has two compensated substrates c1 and c2 and two compensated products c3 and c4. For the compensated species, the elasticities are:

$$\varepsilon_{f, c1}=1$$

$$\varepsilon_{b, c1}=0$$

$$\varepsilon_{f, c2}=1$$

$$\varepsilon_{b, c2}=0$$

$$\varepsilon_{f, c3}=0$$

$$\varepsilon_{b, c3}=1$$

$$\varepsilon_{f, c4}=0$$

$$\varepsilon_{b, c4}=1$$

**Kinetic mechanism:** IRRX

It is assumed that the kinetic mechanism from each of substrates (precursors for the biomass) to the biomass follows the irreversible Michelis-Menten kinetics:

$$v=v_{\max}\frac{s_{1}}{1+s_{1}}$$

where $s_{1}$ represents the scaled concentration of substrates mentioned above. The elasticities can then be expressed as

$$\varepsilon_{f,s_{1}}=\frac{1}{1+s_{1}}$$

$$\varepsilon_{b,s_{1}}=0$$
